# Recommendations for the diagnosis and management of eosinophilic esophagitis in adults and children in Canada: a Delphi consensus project

**DOI:** 10.1093/jcag/gwaf022

**Published:** 2025-11-06

**Authors:** Vishal Avinashi, Milli Gupta, Beth A Payne, Haneen Amhaz, Alisha T Temirova, Waqqas Afif, Dhandapani Ashok, Janice Barkey, David Burnett, Jonathan W Bush, Scott Cameron, Stuart Carr, Dina El Demellawy, Stephanie Erdle, Hien Q Huynh, Jennifer Griffin, Samir C Grover, Kelly Grzywacz, Samira Jeimy, Hin Hin Ko, Gina Lacuesta, Margaret Marcon, Serge Mayrand, Harrison Petropolis, David Rodrigues, Mary Sherlock, Christine Song, Natacha Tardio, Timothy K Vander Leek, Meagan Vurzinger, Brock Williams, Ted Xenodemetroupolous, Christopher Ma, Edmond S Chan

**Affiliations:** Division of Gastroenterology, Hepatology and Nutrition, BC Children's Hospital, Department of Pediatrics, University of British Columbia, V6H 3V4, Canada; Divisions of Gastroenterology and Hepatology, Department of Medicine, Cumming School of Medicine, University of Calgary, Calgary, Alberta, T2N 4Z6, Canada; Women’s Health Research Institute, BC Children’s Hospital Research Institute, Simon Fraser University School of Medicine, V5A 1S6, Canada; Institute for Global Health, BC Children’s Hospital Research Institute, Provincial Health Services Authority, V5Z 2X8, Canada; BC Children’s Hospital Research Institute, V5Z 2X8, Canada; Division of Gastroenterology and Hepatology, McGill University Health Center, McGill University, H4A 3J1, Canada; Division of Pediatric Gastroenterology, Hepatology & Nutrition, Department of Pediatrics, Western University, London, Ontario, N6A 5W9, Canada; Division of Pediatric Gastroenterology, Hepatology & Nutrition, Department of Pediatrics, University of Ottawa, CHEO, Ottawa, Ontario, K1H 8L1, Canada; Division of Pediatric Gastroenterology and Nutrition, Department of Pediatrics, Dalhousie University, B3K 6R8, Canada; Department of Pathology and Laboratory Medicine, University of British Columbia, BC Children’s and Women’s Hospital, V6H 3V4, Canada; Division of Allergy & Immunology, Department of Pediatrics, University of British Columbia, V6H 3N1, Canada; Snö Asthma & Allergy, Abu Dhabi, Abu Dhabi, United Arab Emirates; Department of Pathology, University of Ottawa Ontario, K1Y 4E9, Canada; Division of Allergy, Department of Pediatrics, University of British Columbia, BC Children’s Hospital, Vancouver, British Columbia, V6H 3N1, Canada; Division of Pediatric GI Nutrition, Department of Pediatrics, Stollery Children’s Hospital, University of Alberta, Edmonton Clinic Health Academy (ECHA), Edmonton, Alberta, T6G 1C9, Canada; Division of Pediatric Gastroenterology, Hepatology and Nutrition, Winnipeg Children’s Hospital, Department of Pediatrics, University of Manitoba, R3A 1S1, Canada; Division of Gastroenterology, Scarborough Health Network Research Institute, University of Toronto, Toronto, M1P 2V5, Canada; Division of Pediatric Gastroenterology Hepatology and Nutrition, CHU Sainte Justine, H3T 1C5, Canada; Division of Clinical Immunology and Allergy, Department of Medicine, Western University, Lawson Health Research Institute, London, Ontario, N6A 4V2, Canada; Division of Gastroenterology & Hepatology, Department of Medicine, St. Paul’ s Hospital, University of British Columbia, V6Z 1Y6, Canada; Department of Medicine, Dalhousie University, Halifax Allergy and Asthma Associates, B3H 1X5, Canada; Hospital for Sick Children, Department of Pediatrics, University of Toronto, Toronto, Ontario, M5G 1X8, Canada; Division of Gastroenterology and Hepatology, McGill University Health Center, McGill University, H4A 3J1, Canada; Department of Medicine, Division of Digestive Care & Endoscopy, Dalhousie University, B3H 4R2, Canada; Division of Gastroenterology, Queen's University, K7L 2V7, Canada; Department of Pediatrics, McMaster University, Division of Pediatric Gastroenterology, McMaster Children’s Hospital, L8L 0A4, Canada; University of Toronto, St Michael’s Hospital, Sunnybrook Health Sciences Centre, M5B 1W8, Canada; Adult Clinical Immunology and Allergy, McGill University Health Centre, H4A 3J1, Canada; Pediatric Allergy & Clinical Immunology, Department of Pediatrics, University of Alberta, T6G 1C9, Canada; Division of Gastroenterology, Hepatology and Nutrition, BC Children's Hospital, Department of Pediatrics, University of British Columbia, V6H 3V4, Canada; Division of Allergy, Department of Pediatrics, The University of British Columbia, BC Children’s Hospital Research Institute, V6H 3V4, Canada; Department of Medicine, Division of Gastroenterology and Division of Education and Innovation, McMaster University and Hamilton Health Sciences, L8N 3Z5, Canada; Division of Gastroenterology & Hepatology, Departments of Medicine & Community Health Sciences, University of Calgary, T2N 4Z6, Canada; Division of Allergy, Department of Pediatrics, University of British Columbia, BC Children’s Hospital, Vancouver, British Columbia, V6H 3N1, Canada

**Keywords:** EoE, eosinophilic esophagitis, Delphi, consensus, guideline

## Abstract

**Background:**

Eosinophilic esophagitis (EoE) is a chronic inflammatory disease of the esophagus that effects both pediatrics and adult patients in Canada and is increasing in prevalence. No Canadian focused best practice recommendations currently exist to guide clinical practice.

**Methods:**

The study used a modified Delphi technique to develop evidence and expert opinion-based recommendations for providing care for patients with EoE. The Delphi process consisted of 3 rounds of quantitative surveys and qualitative consensus meetings. Experts were included in the Delphi if they had experience caring for EoE patients in Canada within one of the following professional groups: allergist, adult gastroenterologists, pathologists, pediatric gastroenterologists, and dieticians.

**Results:**

Delphi rounds were completed between May 1, 2024, and June 30, 2024. A total of 31 experts in EoE care from across Canada were recruited to participate in the Delphi consensus process. All participants completed all 3 rounds of Delphi surveys. The final statement includes 38 recommendations for the care of patients with EoE organized into 3 sections: definition, diagnosis, and management. A table of research gaps is provided to stimulate further knowledge development on this topic.

**Conclusion:**

This consensus statement includes actionable recommendations to support quality care of patients with EoE at any age across Canada. We encourage EoE centres in Canada to come together in a multidisciplinary form to not only provide clinical care but also do much needed research on Canadian specific topics and gaps in EoE care.

## Introduction

Eosinophilic esophagitis (EoE), first recognized in the 1990s, is a chronic inflammatory disease of the esophagus, characterized histologically by eosinophil-predominant inflammation in the esophageal mucosa and clinically by esophageal dysfunction.^1–4^ EoE shares pathophysiological characteristics with other type 2 inflammatory diseases such as asthma, atopic dermatitis, and chronic rhinosinusitis with nasal polyps. Prompt diagnosis and treatment are necessary to avoid potential fibrotic complications that can have long-term negative impacts on the patient’s quality of life.^5^ EoE is seen in both children and adults and the incidence and prevalence has been increasing over the past decades.^3^

There have been various national and international clinical practice guidelines published in the past decade;^6–10^ however, the diagnosis and management of EoE continues to evolve as knowledge of the pathophysiology and natural history of the disease increases. There are no existing guidelines for Canada. Canadian healthcare is uniquely delivered in a single-payer, publicly funded system. However, medication coverage occurs through different avenues, including private insurance. The unique Canadian geography coupled with a concentration of specialty care in urban centres, creates additional challenges for the provision of EoE care in Canada that have not been previously addressed. This Canadian focused consensus assesses these concerns and provides recommendations for diagnosis, management and treatment of EoE across all age groups. It is intended to be a practical tool for Canadian clinicians caring for these patients, both in urban and rural centres. Development of the recommendations incorporated both evidence-based literature review, interdisciplinary expert knowledge and experience of EoE care by experts in Canada using a modified Delphi consensus process. This methodology was chosen rather than a GRADE analysis for development of the consensus statement given the limited yet rapidly evolving body of evidence to support clinical care for EoE patients in Canada.

## Methods

### Recommendation development process

This study employed a modified Delphi consensus process and was not registered. The Delphi technique is characterized by a series of rounds that ask for the opinions of experts on a particular topic. This is an evolving process as each round builds on findings from the previous round resulting in a final consensus on the topic of interest. This method is considered an effective strategy when developing tools, guidelines, competencies, anything that requires the knowledge and experience of experts within a field where supportive literature may be limited.^11^ The Delphi process for this study consisted of 3 rounds of iterative anonymous voting with feedback review that utilized both a quantitative survey and qualitative expert consensus group meetings.

A GRADE analysis of the literature was not completed as this paper is not intended as a formal guideline, rather an actionable set of clinical recommendations that reflect current best practice. In addition, a GRADE review of evidence was already complete by the AGA task force in 2020 demonstrating very low quality of evidence and many conditional recommendations, illustrating limited evidence for guideline items. There is a plan to update this document as new literature is published and a hope to move to a formal and evidence-based guideline when more conclusive research is available.

Delphi participants included currently practicing allergists, adult and pediatric gastroenterologists, pathologists, and dietitians who have experience with EoE care in Canada. Recommendations were included in the consensus statement once they met a predefined consensus threshold. Consensus was defined based on 2 primary quantitative criteria: (1) at least 80% agreement (rating of 7 or above) on the Likert scale responses on the survey; and (2) a Kappa score >0.61. After each survey was complete, statements that did not meet quantitative consensus criteria or that had comments recommending rewording were reviewed during a virtual consensus group meeting, reworded and then included in the subsequent round survey for further assessment. This cycle of survey and consensus meeting continued until all expert group members agreed to the final wording of each included statement. Research ethics board approval was obtained for the study from the BC Children’s and Women’s Hospital Research ethics board (H24-00065). Full details of methods are available in Appendix S1.

## Results

**Figure 1.**
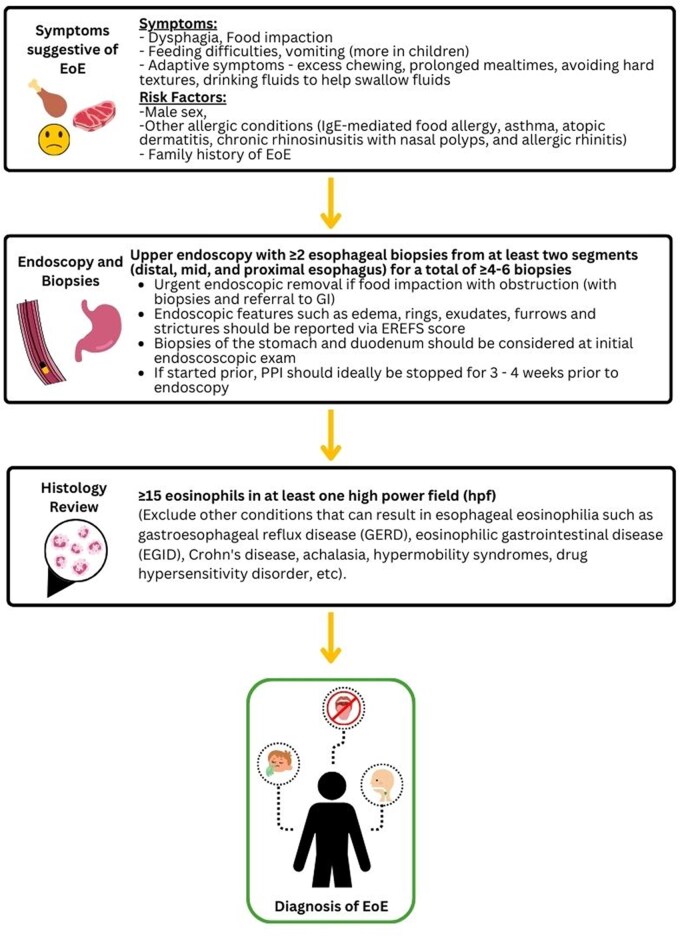

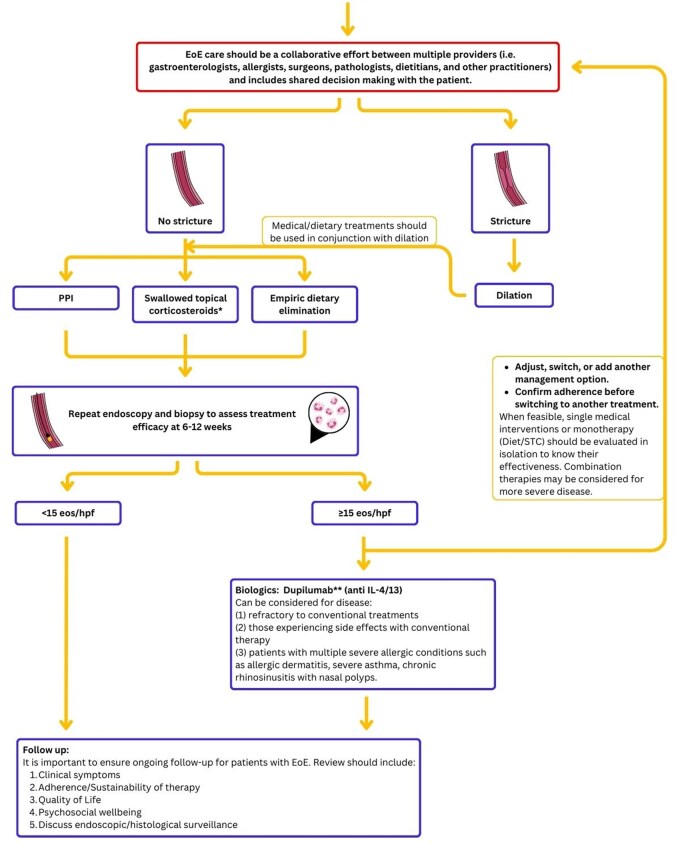
Flow diagram of EoE diagnosis and management. *Budesonide orodispersible tablet (JorvezaR) approved by Health Canada. **Dupilumab (DupixentR) approved by Health Canada.

Three rounds of Delphi voting were completed between May 1, 2024, to June 30, 2024. A total of 31 experts in EoE care from across Canada were recruited to participate in the Delphi consensus process. All participants completed all 3 rounds of Delphi surveys (Table S1). Full details of the results are provided in Tables S2-S4. After the 3 rounds of Delphi consensus building, the final statement includes 38 recommendations for the care of patients with EoE. It was organized into 3 sections for ease of reading: definition, diagnosis, and management. A summary of these recommendations can be found in Table 1, and a clinical flow diagram highlighting critical activities on the path to diagnosis and subsequent management is provided in Figure 1.

**Table 1.** Summary table of all the statements and level of agreement at consensus. Agreement defined as percent ranking statement as 7 or above on Likert scale survey response.

Section	Recommendation	Agreement *n* (%)
**Definition**	Statement #1: EoE is a chronic inflammatory condition of the esophagus, characterized by eosinophilic inflammation. Pathophysiologic mechanisms include barrier dysfunction, delayed (non-IgE mediated) allergic responses to food (and/or environmental allergens), type 2 inflammation, and tissue remodeling.	31 (100)
	Statement #2: EoE is increasing in prevalence, which can be partially attributed to increased awareness. The true prevalence is not well defined in Canada.	29 (93.5)
	Statement #3: EoE occurs in all genders and ethnic backgrounds but is more commonly diagnosed in white males. It is often associated with allergic conditions such as IgE-mediated food allergy, asthma, atopic dermatitis, chronic rhinosinusitis with nasal polyps, and allergic rhinitis. EoE can be associated with family history of EoE in first-degree relatives.	28 (90.3)
	Statement #4: Long-term complications of EoE include fibrosis and stricture development. Risks for these complications are increased with diagnostic delay and untreated EoE.	31 (100)
**Diagnosis**	Statement #5: EoE is defined clinically by symptoms of esophageal dysfunction together with esophageal biopsies showing ≥15 eosinophils/hpf.	28 (90.3)
	Statement #6: Common EoE symptoms in adolescents and adults include dysphagia and food impaction. Adaptive behaviours are frequently seen including drinking fluids to help swallow foods, cutting food into small pieces or pureeing, excessive chewing, prolonged mealtimes, avoiding hard textures, and turning away tablets/pills.	29 (93.5)
	Statement #7: Common EoE symptoms and signs in younger children additionally to those listed in Statement #6, include feeding difficulties, abdominal pain, failure to thrive, and vomiting.	29 (93.5)
	Statement #8: Severe symptoms such as food impaction with obstruction (inability to swallow secretions) need urgent care for endoscopic removal of food bolus. Taking esophageal biopsies at the same time as endoscopy is strongly encouraged. If food bolus impaction passes without need for emergency gastroscope, patients should be referred to GI for either new consultation or a follow-up appointment for endoscopic evaluation.	29 (93.5)
	Statement #9: In situations where access to GI is delayed or unavailable, for example in rural and remote regions of Canada, general surgeons may provide access to endoscopy and biopsy for EoE diagnosis. This endoscopy should follow the same standards outlined in this guideline. Postendoscopy, surgeons may collaborate with the allergist and GI to coordinate management.	29 (93.5)
	Statement #10: A trial of proton pump inhibitors (PPIs) is not required for diagnosis of EoE.	29 (93.5)
	Statement #11: Endoscopic findings for EoE can include Edema, Rings, Exudates, linear Furrows, and Stricture. Finding should be reported as EREFS including subscores. Macroscopic features are absent in some patients with increased eosinophils and are not required to make a diagnosis of EoE.	31 (100)
	Statement #12: Endoscopic biopsies are required for the diagnosis of EoE, regardless of endoscopic appearance. There should be ≥2 biopsies per level from at least 2 segments (distal, mid, and proximal esophagus), for a total of ≥4-6 biopsies. Biopsies of the stomach and duodenum should be considered at initial endoscopic exam.	28 (90.3)
	Statement #13: Histology should be reported as eosinophil counts per 0.3 mm^2^ (=per hpf), with EoE diagnosis requiring ≥15 eosinophils/hpf.	31 (100)
	Statement #14: Classic histologic features beyond esophageal eosinophilia can include dilated intercellular spaces, basal zone hyperplasia, eosinophilic abscesses as well as lamina propria fibrosis, and can strengthen a diagnosis.	31 (100)
	Statement #15: The differential diagnosis of EoE includes gastroesophageal reflux disease, eosinophilic gastrointestinal disease, Crohn’s disease, achalasia, hypermobility syndromes, drug hypersensitivity disorder, and others.	30 (96.8)
**Management**	Statement #16: EoE care should be a collaborative effort between multiple providers (ie, gastroenterologists, allergists, surgeons, pathologists, dietitians, and other practitioners) and includes shared decision-making with the patient.	28 (90.3)
	Statement #17: It is important to transition care from paediatric to adult services to support patients through this high-risk period.	29 (93.5)
	Statement #18: Initial management options for controlling symptoms, reducing inflammation, and preventing complications include PPI, empiric dietary elimination, and swallowed topical corticosteroids. If dilation is required, it must be used as complementary to other treatments as it will not control underlying inflammation.	31 (100)
	Statement #19: Empiric dietary elimination is best started with 1 (cow’s milk) or 2 (cow’s milk and wheat) food elimination, balancing efficacy, convenience, and adherence. Starting with a 6-food elimination may result in higher rates of remission but is not recommended as first-line treatment due to limited convenience, poor adherence, impaired quality of life, and other adverse outcomes.	29 (93.5)
	Statement #20: Consultation with a dietitian is essential for patients on dietary elimination.	31 (100)
	Statement #21: An elemental diet is rarely recommended for management of EoE due to poor taste, frequent need for enteral tube, high cost, and significant impact on quality of life.	27 (87.1)
	Statement #22: Patients with allergic conditions who have undergone prolonged and extensive dietary elimination for the treatment of EoE are at a heightened risk of developing IgE-mediated food allergies to the specific foods they have avoided.	28 (90.3)
	Statement #23: Mode of medication delivery is a shared decision-making process between patient and provider to ensure adherence but prescribers should recognize that patients with EoE frequently have challenges with pills such as tablets and capsules.	30 (96.8)
	Statement #24: Systemic or long-term corticosteroids (eg, oral prednisone) are not recommended for routine use in treatment of EoE.	28 (90.3)
	Statement #25: Budesonide orodispersible tablet is approved by Health Canada for the treatment of EoE in adults. Off-label swallowed topical corticosteroid options include viscous budesonide and fluticasone MDI.	30 (96.8)
	Statement #26: Dupilumab (anti IL-4/13) is a Health Canada approved biologic for EoE and can be considered for disease (1) refractory to conventional treatments; or (2) those experiencing side effects with conventional therapy; (3) and/or patients with currently approved concurrent severe allergic conditions.	25 (80.6)
	Statement #27: The cost of Health Canada-licensed EoE medications can be high. Due to variable coverage by third party prescription programs and lack of current coverage by provincial prescription drug plans, a coordinated effort is needed to ensure patients with EoE receive appropriate treatments regardless of location.	31 (100)
	Statement #28: Development of luminal narrowing and/or stricture can be a complication of EoE. Some narrowing may respond to medical treatment while others may require dilation. While endoscopic dilation may alleviate symptoms, it is important to address mucosal inflammation and prevent recurrence of narrowing through treatment with anti-inflammatory therapies.	29 (93.5)
	Statement #29: Allergy testing (skin prick tests, sIgE blood tests, or patch tests) to uncover food triggers of EoE is not recommended. Rather, if chosen, dietary elimination should be done empirically. The purpose of food allergy testing is to rule out potentially anaphylactic IgE-mediated food allergy when the history is suggestive of it.	30 (96.8)
	Statement #30: In addition to shared understanding and experience in the pathophysiology and management of EoE with gastroenterologists, allergists have unique experience in their ability to manage concurrent and complicating allergic conditions, such as IgE-mediated food allergy and determination of relevant aeroallergen sensitization.	28 (90.3)
	Statement #31: A subset of patients sensitized to pollen may experience seasonal intensification of EoE due to pollen allergy. Allergists can help patients distinguish between EoE and Pollen Food Allergy Syndrome to better manage symptoms.	29 (93.5)
	Statement #32: It is unclear whether sublingual or oral immunotherapy causes or unmasks EoE, or whether the disease is simply associated with the therapy. Immunotherapy should be based on weighing benefits/risks and shared decision-making.	28 (90.3)
	Statement #33: When feasible, single medical interventions or monotherapy (Diet/STC) should be evaluated in isolation to know their effectiveness. Combination therapies may be considered for more severe diseases.^a^	28 (90.3)
	Statement #34: Clinical evaluation alone is not sufficient to assess treatment efficacy. Repeat endoscopy and biopsy to assess treatment efficacy after a change in management ideally should occur at 6-12 weeks.	27 (87.1)
	Statement #35: It is important to ensure follow-up of EoE. The interval for follow-up including endoscopy may vary depending on symptoms (frequency and severity), phenotype including history of strictures, and amount of inflammation, with uncontrolled EoE requiring more frequent follow-up.	28 (90.3)
	Statement #36: Disease activity indices for symptomatic, endoscopic, histologic, and quality-of-life measures are available and can be used, although may not always be feasible to adopt in routine clinical settings.	28 (90.3)
	Statement #37: EoE has a negative impact on psychosocial status. Monitoring the overall well-being of EoE patients is an important part of follow-up.	30 (96.8)
	Statement #38: The ultimate duration of therapy for patients who achieve control of their EoE is unclear in the literature. Given this is a long-term condition, the decision to continue treatment and in what form is dependent on severity of symptoms and disease, as well as shared decision-making with the patients and family; balancing risk and benefits of the treatment with the risks of complications (eg, fibrostenotic disease).	28 (90.3)

a EoE = Eosinophilic Esophagitis; IgE = Immunoglobulin E; hpf = high powered field; GI = gastroenterology; PPI = Proton Pump Inhibitor; MDI = Metered-Dose Inhaler; IL = Interleukin; sIgE = specific immunoglobulin E; STC = swallowed topical corticosteroids;

### Final consensus statements with evidence summaries

#### Section 1: Definition

**Statement #1: EoE is a chronic inflammatory condition of the esophagus, characterized by eosinophilic inflammation. Pathophysiologic mechanisms include barrier dysfunction, delayed (non-IgE mediated) allergic responses to food (and/or environmental allergens), type 2 inflammation and tissue remodelling.**

The pathophysiology of EoE is not fully understood and involves a complex interplay between genetic, environmental, antigenic, and intrinsic factors. The disease is chronic and progressive, involving allergen-induced, type 2 immune activation of the esophageal epithelium leading to mucosal inflammation, barrier dysfunction, and eventual tissue remodeling and organ dysfunction.^12^ The inflammatory response is thought to be a result of stimulation of (non-IgE) immune cells in the esophagus by antigens releasing inflammatory cytokines that drive eosinophil recruitment.^13^ In addition, the esophageal epithelium of an EoE patient has impaired barrier function that increases permeability to allergens which can trigger further disease.^12^^,^^14^ Patients with EoE are at high-risk of associated IgE-mediated allergic conditions, as described in statement #3. Clinicians should be aware that this association is bi-directional.

**Statement #2: EoE is increasing in prevalence, which can be partially attributed to increased awareness. The true prevalence is not well defined in Canada.**

A recent systematic review and meta-analysis found the pooled EoE incidence rate was 3.7/100 000 persons/year [95% confidence interval (CI): 1.7-6.5] and was higher for adults (7; 95% CI: 1-18.3) than for children (5.1; 95% CI: 1.5-10.9). The pooled prevalence of EoE was 22.7 cases/100 000 inhabitants (95% CI: 12.4-36). Prevalence was higher in adults than in children (43.4; 95% CI: 22.5-71.2 vs 29.5; 95% CI: 17.5-44.7, respectively), in American compared to European studies.^15^ There is limited data from Canada. A study from Edmonton, Alberta, suggests recent pediatric incidence is much higher at 11.1 cases per 100 000 between 2015 to 2018.^16^ Rates of disease are increasing in Canada and globally. An evaluation of time trends in EoE incidence in Calgary, Alberta over 15 years demonstrated a 50.2% yearly increase in incidence that could not be explained by changes in diagnostic practice alone, suggesting there are other factors than increased awareness and testing driving this increase.^17^ Similar findings have been presented across North America and Europe, with increasing rates of diagnosis ranging from 2- to 100-fold per year, outpacing the increase seen in rates of biopsy and endoscopy in these regions.^18–21^

**Statement #3: EoE occurs in all genders and ethnic backgrounds but is more commonly diagnosed in White males. It is often associated with allergic conditions such as IgE-mediated food allergy, asthma, atopic dermatitis, chronic rhinosinusitis with nasal polyps and allergic rhinitis. EoE can be associated with a history of EoE in first-degree relatives.**

Epidemiological data from Canada and globally suggests White males are between 2 to 10 times more likely to be diagnosed with EoE than females.^15^^,^^22–24^ Additional evidence for increased likelihood of diagnosis in White populations is supported by a Canadian study which found a paucity of East Asian (including Chinese and Japanese) pediatric patients, compared with White and South Asian patients, in an EoE cohort.^25^ Association with type 2 inflammatory^26^ or allergic conditions^27^ and family history (of known EoE and/or dysphagia) is well documented.^28^

**Statement #4: Long-term complications of EoE include fibrosis and stricture development. Risks for these complications are increased with diagnostic delay and with untreated EoE.**

Esophageal inflammation in the setting of EoE leads to tissue remodeling with fibrosis and probable stricture formation.^3^ The risk of stricture is estimated to increase by 9% for every year that EoE goes untreated, and is associated with increasing risk of food impaction too. In cohort studies if the delay in diagnosis was greater than 10 years, the estimated risk of stricture was >40%.^29^^,^^30^ It is important to note that the occurrence of EoE symptoms does not necessarily correlate with disease severity. EoE can occur with mild symptoms and/or symptoms of well adapted eating behaviours, which are often unrecognized and contribute to diagnostic delays.^3^ A Canadian pediatric study found that 4% of pediatric cases developed stricturing and 12% had more subtle narrowing,^16^^,^^31^ highlighting the long-term complications of EoE can occur in pediatric patients. There has been a growing interest to study the consequences of chronic inflammation by functional luminal imaging probe planimetry. This is a balloon catheter device that uses impedance planimetry to assess for stiffness and distensibility in the esophageal wall by evaluating changes in diameter with balloon pressure.^32^^,^^33^ It holds promise but its clinical availability is limited due to resources and cost.

#### Section 2: Diagnosis

**Statement #5: EoE is defined clinically by symptoms of esophageal dysfunction together with esophageal biopsies showing ≥15 eosinophils/hpf.**

Diagnostic criteria have previously been established through international consensus with the most recent revisions from the 2018 AGREE guidelines, and the panel agreed that these criteria are applicable to Canadian patients with EoE. A clinicopathological diagnosis of EoE is made from symptoms of esophageal dysfunction (which can vary with age but classically involve dysphagia, food sticking, vomiting) in addition to ≥15 eosinophils per high power field (eos/hpf) on esophageal biopsy isolated to the esophagus, and assessment of non-EoE disorders that can contribute or cause esophageal eosinophilia.^34^ While the histopathology may have other supportive features beyond peak eosinophil count, they are not a required part of the diagnostic criteria. Similarly, endoscopic features are helpful to describe but not required in making the diagnosis of EoE, as patients can have normal endoscopic appearance in EoE.^34^

**Statement #6: Common EoE symptoms in adolescents and adults include dysphagia and food impaction. Adaptive behaviours are frequently seen including drinking fluids to help swallow foods, cutting food into small pieces or pureeing, excessive chewing, prolonged mealtimes, avoiding hard textures and turning away tablets/pills.**

Symptoms vary with age of onset. In older patients, dysphagia is the most common clinical symptom (>70% of cases)^29^^,^^35^^,^^36^ followed by food impaction (∼30%), which is more commonly seen in men.^36^^,^^37^ Adaptive eating behaviours typically develop as a way of coping with longstanding dysphagia. The exact behaviours are described and best remembered through the acronym “IMPACT”: Imbibing fluids with meals to lubricate foods; Modifying food (cutting into small pieces); Prolonged mealtimes; Avoidance of hard textured foods (eg, bread, meats); Chewing excessively; Turning away pills. Other less common (<60%) adult symptoms can include heartburn, reflux and chest pain.^35^

**Statement #7: Common EoE symptoms and signs in younger children additionally to those listed in statement #6 include feeding difficulties, abdominal pain, failure to thrive, and vomiting.**

As is the case with older patients described above, symptoms present at diagnosis for children can vary with age. Abdominal symptoms such as epigastric pain and reflux are more common when diagnosis occurs before the age of 11^38^^,^^39^ affecting between 30% and 100% of patients.^35^ Dysphagia is seen less often in children than adults (<60%).^35^ Failure to thrive is a unique symptom of pediatric disease most often associated with onset at <6 years.^35^ Feeding avoidance and poor progression of solids can be present and can overlap with pediatric feeding disorders, often presenting as picky eating. Vomiting is often seen in infants and toddlers and is more common in non-White patients.^38^^,^^39^ Many will have concomitant type 2 allergic conditions, which raises the risk profile for EoE.^26^^,^^27^ Therefore, early assessment should be considered in these young patients for the diagnosis of EoE.

**Statement #8: Severe symptoms such as food impaction with obstruction (inability to swallow secretions) need urgent care for endoscopic removal of food bolus. Taking esophageal biopsies at the same time as endoscopy is strongly encouraged. If food bolus impaction passes without need for emergency gastroscope, patients should still be referred to gastrointestinal (GI) for either a new consultation or a follow-up endoscopic evaluation.**

Severe symptoms such as food impaction occur in approximately 16% of EoE patients.^40^ Severe symptoms are more often associated with active inflammation and fibrotic features, such as rings and or strictures, which typically represent disease chronicity. Gastroscopes for removal of food impaction should occur where endoscopy services are available, and sometimes in the emergency department. Methods and techniques for endoscopic bolus removal are beyond the scope of this article; however, collections of biopsies at the time of food bolus removal to confirm diagnosis or monitor disease (if diagnosis is already established) are strongly encouraged (see statement #11). This aligns with other existing guidelines,^41^^,^^42^ but currently is not consistently done in the Canadian landscape at the time of food impaction.^43^

**Statement #9: In situations where access to GI is delayed or unavailable, for example in rural and remote regions of Canada, general surgeons may provide endoscopy and biopsy for EoE diagnosis. This endoscopy should follow the same standards outlined in this guideline. Post-endoscopy, surgeons may collaborate with the allergist and GI to coordinate management.**

In Canada, delayed access to gastroenterology occurs due to current limits in specialist availability, particularly in rural areas. For example, where in Canada a patient lives may determine whether biopsy is done after food impaction (see statement #8). Despite this, every effort should be made to support appropriate management and follow-up as described in these guidelines. General surgeons in Canada can safely facilitate diagnosis, including provision of endoscopy and biopsy to reduce diagnostic delays.^44^ General surgeons who perform endoscopy and biopsy would be expected to follow the same procedural recommendations provided in this consensus statement (see statement #12). Evidence is mixed but does suggest that EoE rates may be higher in rural areas, potentially due to increased exposure to allergens^45^ this is balanced potentially by limited access to specialist care.^46^ Advocacy for increased access to specialist care for rural patients is needed to support the best health outcomes for EoE patients.

**Statement #10: A trial of proton pump inhibitors (PPIs) is not required for diagnosis of EoE.**

Reliance on a trial of PPIs to confirm diagnosis of EoE was previously used to distinguish EoE from gastroesophageal reflux disease (GERD); however, recent research suggests that the interaction between these disorders is much more complex, and PPI can help in EoE via mechanisms that are independent of acid reduction (see statement #18).^47^^,^^48^ Clinical response to PPI does not preclude a diagnosis of EoE and a referral should not be delayed for its evaluation. However, the panel recognizes that many patients are pragmatically trialed on PPI therapy while awaiting results of confirmatory biopsies. For accurate diagnosis of EoE, PPI should be stopped at least 3-4 weeks prior to endoscopy as per recent British and Italian EoE guidelines.^9^^,^^49^

**Statement #11: Endoscopic findings for EoE can include edema, rings, exudates, linear furrows, and stricture. Findings should be reported as EREFS including subscores. Macroscopic features are absent in some patients with increased eosinophils and are not required to make a diagnosis of EoE.**

The most common endoscopic findings in EoE patients are edema (loss of vascular pattern), rings, exudates, linear furrows, and strictures each of which are reported to occur in approximately 20%-50% of cases.^50^^,^^51^ Up to 93%^50^ of EoE patients will exhibit one or more of these findings but up to 21% of patients^51^ can have a normally appearing esophagus. The Eosinophilic Esophagitis Endoscopic Reference Score (EREFS) is a validated grading system that can be used in adult and pediatric populations for assessment of treatment response.^52^ Endoscopic findings are also included in the Index of Severity for Eosinophilic Esophagitis (I-SEE) scoring system. In addition to clinical features including symptom frequency, severity, and presence of complications, I-SEE also includes endoscopic findings included in the EREFS score. These are inflammatory features (edema, furrows and exudates) and fibrostenotic components (rings and strictures) to evaluate disease severity (see statement #14).^53^ I-SEE holds promise as a possible assessment tool in clinical practice, with short term validation studies predicting important outcomes such as need for dilation.^54^

**Statement #12: Endoscopic biopsies are required for the diagnosis of EoE, regardless of endoscopic appearance. There should be ≥2 biopsies per level from at least 2 segments (distal, mid, and proximal esophagus), for a total of ≥4-6 biopsies. Biopsies of the stomach and duodenum should be considered at initial endoscopic exam.**

Significant variability in histologic findings can occur across different biopsy sites in patients with EoE. Therefore, multiple biopsy sites are required to improve diagnostic sensitivity, taken from the most inflamed area seen on endoscopy. In a retrospective study of 66 adults with 341 biopsy specimens, a single biopsy had a sensitivity of 55% based on the ≥15 eos/hpf definition, but increased to 100% when 5 biopsies were taken.^55^ Biopsies of the stomach and duodenum are considered, particularly in pediatric patients or those with other GI symptoms or macroscopic abnormalities, to rule-out non-EoE eosinophilic gastrointestinal diseases (EGID)^34^^,^^56^ and/or other GI diseases such as celiac disease. The expectation for care and collection of biopsies is that biopsies are taken in a manner that ensures adequate sample for diagnosis. Follow-up should be arranged with the endoscopist to review pathology results with the patient.

**Statement #13: Histology should be reported as eosinophil counts per 0.3 mm^2^ (=per hpf), with EoE diagnosis requiring ≥15 eosinophils/hpf.**

This guideline supports a standard for diagnosis of EoE of ≥15 eosinophils in at least 1 hpf from the esophageal biopsies in line with other recently published guidelines.^34^ The use of a standard measure for reporting and diagnosis of eosinophilic density based on ≥15 eos/hpf on esophageal biopsy had a sensitivity of 100% and specificity of 96% in prospective evaluation.^57^ Digitization of the hpf using the method proposed and validated by Dellon et al allows for standardized reporting of eosinophil density per 0.3 mm^2^.^58^

**Statement #14: Classic histologic features beyond esophageal eosinophilia can include dilated intercellular spaces, basal zone hyperplasia, eosinophilic abscesses as well as lamina propria fibrosis, and can strengthen a diagnosis.**

Classic histological features are highly correlated with eosinophil counts,^59^^,^^60^ yet they can be useful in both pediatric and adult patients as a mechanism to support diagnosis and further distinguish EoE from GERD or other related conditions. When feasible, an additional measure of esophageal pathology that can be considered is the EoE Histological Scoring System (EoEHSS).^61^ This scoring system evaluates 8 histologic features of EoE, specifically eosinophilic inflammation, basal zone hyperplasia, eosinophilic abscesses, eosinophilic surface layering, dilated intercellular spaces, surface epithelial alteration, dyskeratotic epithelial cells, and lamina propria fibrosis.

EoEHSS has been shown to outperform use of peak eosinophil counts alone for assessment of treatment status in EoE.^61^^,^^62^ A recent validation of the EoEHSS in Canadian children suggests that the score is valid for prediction of active disease state and can be used for prognosis.^63^ Further research is required to validate model performance prospectively. Basal zone hyperplasia and lamina propria fibrosis have been included as part of the I-SEE,^53^ a composite tool that includes histology, endoscopy and clinical symptoms. It has a high potential to be used in clinical practice.

**Statement #15: The differential diagnosis of EoE includes, GERD, EGID, Crohn’s disease, achalasia, hypermobility syndromes, drug hypersensitivity disorder, and others.**

Despite its increasing recognition, EoE remains underdiagnosed and often misunderstood. Its symptoms can mimic or accompany those of GERD, and other related conditions leading to misdiagnosis and inappropriate treatment or lack thereof. Use of the diagnostic criteria presented in the previous recommendations are critical to ensure appropriate and timely diagnosis. Diagnosis and distinguishing EoE from other related conditions (such as GERD) require consideration of symptoms, access to endoscopic procedures and detailed review of histological findings. There is no standard work up to rule out other conditions given the wide differential, especially for esophageal eosinophilia which can include infections, drug reactions, hypereosinophilic syndrome, auto-immune disorders and more. While EoE is within the EGID spectrum, specific diagnostic criteria for non-EoE EGIDs are out of the scope of this article but further references are available.^64^

#### Section 3: Management

**Statement #16: EoE care should be a collaborative effort between multiple providers (ie gastroenterologists, allergists, surgeons, pathologists, dietitians, and other practitioners) and includes shared decision-making with the patient.**

Effective clinical care for patients with EoE requires input from multiple specialities and the patient (families) themselves. This document supports a collaborative approach like that defined by Sauer et al^65^ as “a collective effort of individuals with diverse expertise who collaborate to develop comprehensive, coordinated medical care and research.” We also support a broad definition of shared decision making as a method of care where the patient is involved from the start in defining the problem and considering the best solution for their individual condition.^66^ Involving a multidisciplinary team means all aspects of the disease can be addressed and requires effective communication amongst providers and the patient. Multidisciplinary team-based care can lead to improved patient outcomes and quality of life in the setting of EoE.^65^^,^^67^

In a prospective study including 243 adults and 270 caregivers of children diagnosed with EoE, shared decision making was associated with significantly increased satisfaction with treatment (odds ratio [OR] = 2.62, 95% CI, 1.76-3.92).^67^ Engaging patients in decision making about their treatment should include a focus on increasing beliefs where personal gain, doubts, and perceived beliefs about necessity of treatment are addressed. Decreased belief in the necessity of treatment is a strong predictor of poor treatment adherence in adult patients with EoE.^68^ A recent qualitative evaluation of all data from 3 conversational electronic health forums for patients and caregivers of patients with EoE between 2018 and 2020 identified themes related to patient perspectives of EoE management. This analysis supported the value of shared decision making as an opportunity to address treatment misconceptions, identify shared and realistic treatment goals and increase belief in treatment necessity.^69^

**Statement #17: It is important to transition care from paediatric to adult services to support patients through this high-risk period.**

Health care transition is important for adolescents with chronic medical conditions, with EoE being no exception. A survey of patients and parents with EoE found a significant deficit in health care transition readiness and knowledge with over 75% of respondents indicating no knowledge of the transition process and expressing the need for tailored support services.^70^^,^^71^ Some highlighted transition concerns include poor communication between providers and a lack of confidence in patients when taking on self-management of their symptoms due to limited disease knowledge.

A lack of transition of care can result in a disjointed care, loss to follow-up,^72^ negative quality of life and poor health outcomes. Transition is not a single event but a process that starts years before transfer to adult care. Coordinated programs involving pediatric and adult practitioners while not always feasible can improve patient outcomes and decrease condition related anxiety.

**Statement #18: Initial management options for controlling symptoms, reducing inflammation, and preventing complications include PPI, empiric dietary elimination, and swallowed topical corticosteroids. If dilation is required, it must be used as complementary to other treatments as it will not control underlying inflammation.**

First line treatments for EoE include pharmacological agents or empiric dietary elimination to reduce inflammation. PPIs provide an anti-inflammatory effect by inhibiting IL-4-stimulated eotaxin-3 expression in EoE esophageal cells and blocking STAT6 binding to the promoter. Proton pump inhibitors, which are typically prescribed at a higher dose, are an accessible and low-cost treatment which improve histology in approximately 50% of patients.^73^ While there is no truly predictive model, 1 study supports an allergic phenotype is associated with a lower likelihood of response to PPI.^74^

Swallowed topical corticosteroids, such as swallowed metered dose inhaler (fluticasone) or budenoside either through a liquid suspension, or an orodisperisble tablet, can be an effective treatment option (see statement #25).^75–79^ The anti-inflammatory mechanism is not fully understood but may including inhibition of pathways involved with molecular signaling including IL-13, improving tight junctions, and decreasing remodeling (fibrosis).^80–82^

The use of dilation can aid in symptom relief but does not deal with the underlying inflammation. Dilation must be used with other effective treatments for long-term improvements. Complications of dilation are rare, and are more likely to occur in a smaller diameter esophagus.^83^^,^^84^

**Statement #19: Empiric dietary elimination is best started with 1 (cow’s milk) or 2 (cow’s milk and wheat) food elimination, balancing efficacy, convenience, and adherence. Starting with a 6-food elimination may result in higher rates of remission but is not recommended as first-line treatment due to limited convenience, poor adherence, impaired quality of life and other adverse outcomes.**

Multiple studies in pediatrics^85–87^ and adults^88^ have evaluated the efficacy of empiric elimination diets and found that the most common causative food allergen is milk (found in ∼40%-50% of cases). Efficacy of dietary therapies, defined based on symptom reduction and achieving <15 eosinophils/high powered field, increases with step-up elimination diets; increasing from 43% with a 3-food elimination; 60% with 4-food; and 79% with 6-food elimination. However, adherence drops with each additional step.^84^ A recent study showed no difference in response for 1 food (dairy) versus 6 food or 1 versus 4 food elimination diet; therefore supporting a single food elimination as a viable treatment option.^86^^,^^89^ Other data supports similar findings for 2-food elimination diets^84^ (dairy and wheat). Step-up elimination strategies reduce time and resource utilization from time from diagnosis to remission by approximately 20%, compared to top-down approaches starting with 6-food elimination diets.^84^ However, the adverse outcome associated with elimination diets, particularly in pediatric populations, include nutritional problems and increased risk of feeding disorders such as avoidant/restrictive food intake disorder (ARFID).^90^

**Statement #20: Consultation with a dietitian is essential for patients on dietary elimination.**

When patients are following a dietary elimination strategy, close follow up from a dietitian is recommended to evaluate nutritional adequacy and dietary adherence. Parents of children undergoing dietary elimination report problematic feeding behaviours. Therefore, there is a need for support, planning and strategizing to manage issues and ease parental stress. Children that undergo dietary elimination need dietary guidance due to their risk of low vitamin D and calcium intake.^91^ Adults can similarly benefit on optimization of micronutrients. Dietitians can support patients and families navigate the complexity of elimination diets by providing advice on meal plans/feeding difficulties, deliver motivation, and encourage commitment to support adherence.

**Statement #21: An elemental diet is rarely recommended for management of EoE due to poor taste, frequent need for enteral tube, high cost and significant impact on quality of life.**

An elemental diet, or amino acid-based diet, has demonstrated high efficacy for histopathological remission in both adults and children(∼90%).^92^^,^^93^ In all studies, drop-out rates ranged from 5% to 20% with the primary reason being unpalatability of the diet.^94–96^ In a study of 164 pediatric patients, 135 (82%) required a nasogastric tube for feeding adherence.^95^ Significant weight loss was observed in adults following elemental diets, which has a negative impact on quality of life.^96^ Elemental diets restrict food options more than elimination diets and negatively impact lifestyle, further reducing compliance when attempted. Elemental diet is not meant to be used as long-term maintenance therapy, which further limits its applicability in routine practice.

**Statement #22: Patients with allergic conditions who have undergone prolonged and extensive dietary elimination for the treatment of EoE are at a heightened risk of developing IgE-mediated food allergies to the specific foods they have avoided.**

Limited evidence from case studies in adults and children with a diagnosis of EoE suggests the risk of severe IgE-mediated food allergies can develop after strict elimination diets from 6 months to over 2 years. In all cases, the patient developed new and often severe (such as anaphylaxis and/or urticaria) reactions to re-introduced foods when that food group was identified as a cause for their EoE.^97–100^ A similar relationship between allergic triggers and development of new severe IgE-mediated food allergy is well established in cases of atopic dermatitis treated with elimination diets. A total of 19% of 298 patients with atopic dermatitis presumed to be triggered by foods developed new immediate food reactions after elimination diets, with 30% of the reactions being anaphylactic.^101^ Therefore, clinicians should carefully monitor patients during food re-introduction, particularly if they have a history of other severe allergic conditions such as atopic dermatitis,^102^ and caution patients accordingly. Engagement of an allergist may be indicated in these cases to support patients through these transitions.

**Statement #23: Mode of medication delivery is a shared decision-making process between patient and provider to ensure adherence, but prescribers should recognize that patients with EoE frequently have challenges with pills such as tablets and capsules.**

Shared decision making is a process that involves clinicians and patients working together to review treatment options, risks and goals for treatment in order to make collaborative decisions on a care plan.^66^ Patients with EoE frequently have challenges swallowing pills as it can be uncomfortable and result in impaction. When possible, clinicians should offer options for the mode of delivery when prescribing medications. If pills are required, consider dissolvable medications or liquids, or find the smallest pill size in the class of medication. The choice of medication formulation must be balanced with other factors, including availability and cost.

**Statement #24: Systemic or long-term corticosteroids (eg, oral prednisone) are not recommended for routine use in treatment of EoE.**

Clinical trial evidence supports this recommendation in pediatric patients, but the principle applies to all EoE patients. A prospective randomized trial including in 80 children found no evidence of a short- or long-term clinical advantage from systemic corticosteroids (oral prednisone) compared to topical (swallowed fluticasone).^103^ This study found rapid initial improvement in clinical and histologic outcomes in both systemic and topical corticosteroids at 4 weeks; with similar rates of remission (∼40%) by week 24 in both groups. Adverse effects (such as hyperphagia, weight gain, cushingoid features) of systemic corticosteroids were observed in 40% of the treatment group and none in the topical steroid group.

**Statement #25: Budesonide orodispersible tablet is approved by Health Canada for the treatment of EoE in adults. Off-label swallowed topical corticosteroid options include viscous budesonide and fluticasone Metered-Dose Inhaler (MDI)**.

Budesonide orodispersible tablets (BOT) have been evaluated in placebo controlled randomized trials for both initial and maintenance therapy leading to Health Canada approval in adults but not pediatrics. These trials provide high-quality evidence that BOT treatment can lead to both symptom and histological improvement over long time periods. In a trial of 88 adults with histologically confirmed EoE, 58% achieved remission of clinical and histological symptoms 6 weeks after therapy initiation compared to none in the placebo group. Remission rate improved to 85% when therapy was extended to 12 weeks using a 1 mg BOT twice daily.^104^ In a trial of 204 adults who had already achieved remission with 1 mg BOT twice daily for at least 6 weeks, remission was further maintained in 75% of participants for up to 48 weeks. Adverse events reported were similar in both groups. A lower dose of 0.5 mg BOT twice daily maintained clinicohistological remission in 73.5% of cases over a similar period. Remission was defined based on absence of symptoms of dysphagia and odynophagia on each day for the last week of treatment and peak eosinophil count <15 eosinophils/mm^2^ hpf.^105^ A further extension of BOT treatment up to 3 years still supports high remission rates with no new safety concerns.^106^ The FDA approved liquid Budesonide formulation, Eohilia,^107^ has not undertaken approval by Health Canada. Alternative off-label treatments including swallowed topical corticosteroids from metered inhalers or viscous solutions. These are commonly used for both adults and pediatrics, with demonstrated evidence of effectiveness in multiple clinical trials for histological remission.^75–79^ As an oral suspension, budesonide is mixed with a viscous vehicle, such as Splenda (R), maple syrup, applesauce, or honey, to create a slurry that is swallowed, allowing the medication to coat the esophagus and reduce inflammation.^108^ After administration of the medication, food and drink should be avoided for at least 30 minutes. There is a risk of esophageal candidiasis with the use (<5%) and a *theoretical* risk of adrenal insufficiency, especially if the patient is on numerous other topical steroids. Rinsing the mouth after medication administration is suggested to decrease the risk of esophageal candidiasis. Prescribing frequency (such as daily vs BID dosing) should strike a balance between potential effectiveness and adherence.

**Statement #26: Dupilumab (anti IL-4/13) is a Health Canada approved biologic for EoE and can be considered for disease (1) refractory to conventional treatments; or (2) those not tolerating conventional therapy; (3) and/or patients with currently approved concurrent severe allergic conditions.**

Dupilumab, a fully human monoclonal antibody, blocks interleukin-4 and interleukin-13 signaling, which have key roles as drivers of type 2 inflammation in EoE. Dupilumab is the first biologic agent approved by Health Canada for use in both adults (May 2023) and pediatrics (1 year old and older) (September 2024) for the treatment of EoE. It was approved based on clinical trial evidence from a large, multinational phase 3 trial with 3 parts.^109^ Part A included 81 patients with diagnosed EoE over 12 years of age randomized (1:1) to either 300 mg subcutaneous Dupilumab weekly or placebo for 24 weeks followed by both groups receiving weekly 300 mg subcutaneous Dupilumab up to 52 weeks post treatment initiation regardless of initial study arm (phase 3). Part B involved randomization (1:1:1) of 240 patients to either 300 mg subcutaneous Dupilumab weekly or every 2 weeks compared to placebo for 24 weeks. After 24 weeks (until 52 weeks) Part C participants continued either 300 mg subcutaneous Dupilumab weekly or every 2 weeks, including those who had originally been in the placebo arm. This trial concluded that weekly dosage of Dupilumab was effective at achieving clinicohistological remission, whereas every 2-week dosing achieved histological remission but not symptom control. Adverse events were similar across groups.^110^ These results are similar to previous phase 2 trials.^111^^,^^112^ Phase 3 trial in pediatric patients 1 to 11 years of age randomized participants between higher and lower exposure groups (based on body weight) concluded that Dupilumab was effective at achieving histologic remission at week 16 for both doses, but only the higher dose resulted in improvement of key secondary end points (endoscopic, transcriptomic metrics).^113^ A limitation of the pediatric study was the finding that there were no significant differences in symptoms reported in any group, which the authors attributed to age-related limitations in cognitive and verbal development as well as the adoption of compensatory behaviours.

Additional advanced therapies, such as mepolizumab, reslizumab, and benralizumab (interfering with IL-5 axis), cendakimab and dectrekumab (anti-IL-13s), tezepelumab (anti-TSLP), etrasimod (S1P receptor modulator), lirentelimab (anti-SIGLEG-8), JAK inhibitors have all been identified as potential drug targets for future therapies. Barzolvolimab (CDX-0159)^114^ and human alpha-1-proteinase inhibitor^115^ are currently under phase 2 study and may be approved in the future.^116^

**Statement #27: The cost of Health Canada-licensed EoE medications can be high. Due to variable coverage by third party prescription programs and lack of current coverage by provincial prescription drug plans, a coordinated effort is needed to ensure patients with EoE receive appropriate treatments, regardless of location.**

The primary Health Canada approved pharmacological treatments for EoE, BOT and Dupilumab are restricted in their accessibility through limited reimbursement criteria. For example, not all private or public health insurance plans provide long-term coverage for BOT, which is often only reimbursed for induction of remission over a 6-week treatment course.^117^ Beyond 6 weeks, use is either for extended induction in delayed responders or for maintenance therapy, which currently often requires payment out-of-pocket payments.

Health Canada approval of Dupilumab for the treatment of EoE was obtained in 2023. However, at the time of this consensus, review at Canada’s Drug Agency for public reimbursement for the EoE indication had not been pursued. Public coverage for dupilumab for other indications of type 2 inflammatory diseases such as asthma, atopic dermatitis, chronic rhinosinusitis with nasal polyps is available. It should be noted that dosing (and associated costs) of dupilumab for EoE (weekly dosing) is double that of other indications, where dosing is bi-weekly. Other off-label treatments of swallowed topical corticosteroids are currently not approved for insurance coverage.

The Government of Canada has recognized the challenges faced by the public and the healthcare system because of costly drugs and proposed initial legislation to establish a national Pharmacare plan.^118^ Although this may be a great advancement for health equity, it does not address therapies for EoE and as such, advocacy for financial support for patients (either directly through pharmaceutical companies or with insurers) is often required. This advocacy work can result in additional burden for the patient, family, physicians, and care providers.

**Statement #28: Development of luminal narrowing and/or stricture can be a complication of EoE. Some narrowing may respond to medical treatment while others may require dilation. While endoscopic dilation may alleviate symptoms, it is important to address mucosal inflammation and prevent recurrence of narrowing through treatment with anti-inflammatory therapies.**

Strictures occur in approximately 15% (reports ranging from 13% to 70%) of cases of EoE in adults and 8% (reports ranging from 0.2% to 28%) in children with rising risk if treatment is delayed.^30^^,^^50^^,^^95^^,^^119^ In a retrospective analysis of 200 adult EoE cases (median age of diagnosis 39.5 years), the prevalence of esophageal strictures increased significantly from 17.2% to 70.8% with increased diagnostic delay (diagnostic delay 0-2 years compared to >20 years; *P* < .001). A delay in diagnosis was the primary risk factor for strictures at the time of EoE diagnosis (OR = 1.08; 95% CI, 1.040-1.122; *P* < .001 for every year delayed).^30^ Diagnostic delay was greatest in patients with symptom onset at <20 years of age, indicating vigilance in this age group to reduce complications.^30^ In a meta-analysis of trials including 27 studies (*n* = 845 adults; *n* = 87 pediatric) with median number of dilations of 3 per patient, endoscopic dilation resulted in symptom improvement in 95% of patients and major complications occurred in less than 1% of patients.^120^ Symptom improvement with dilation has not been shown to translate to underlying disease improvement based on inflammatory measures.^121^ Therefore, medical/dietary treatments should be used (eg, PPIs, swallowed topical corticosteroids, empiric elimination diets and/or biologics) in conjunction with dilation to reduce patient symptom burden.^122^

**Statement #29: Allergy testing (skin prick tests, sIgE blood tests, or patch tests) to uncover food triggers of EoE is not recommended. Rather, if chosen, dietary elimination should be done empirically. The purpose of food allergy testing is to rule out potentially anaphylactic IgE-mediated food allergy when the history is suggestive of it.**

Despite evidence demonstrating poor predictive value of allergy tests for identifying EoE food triggers, many patients, families, and clinicians continue to demand such a work-up.^123^^,^^124^ Early research on this topic from a single centre suggested allergy testing may be useful for uncovering EoE food triggers, but the results were not reproducible.^125^ Since then, multiple research studies and a systematic review with meta-analysis have confirmed allergy tests (skin prick tests, sIgE blood tests, or patch tests) are poorly predictive of EoE food triggers. In fact, effectiveness (∼45.5%) of identifying food triggers by skin prick testing is lower than empiric dietary elimination (see statement #19).^93^^,^^126–129^ Thus, EoE clinical practice guidelines recommend against allergy testing to elicit EoE food triggers.^9^^,^^49^ Although IgE-mediated food allergy and EoE can co-exist (those with IgE-mediated food allergy can have a 9.1 fold higher risk of subsequent EoE),^27^ the pathophysiology of EoE (see statement #1) is that of delayed non-IgE-mediated food responses, providing a plausible mechanistic rationale for why skin prick and sIgE testing should be reserved for confirmation of potentially anaphylactic IgE-mediated food allergy and not EoE food triggers.^130^ This explanation supports why biologics targeting non-IgE mechanisms have been found to be more effective than those directly targeting IgE antibodies.^131^ Repeated medical education of patients and practitioners is required to change practice patterns that demand “panel testing.”^132^ The risks of panel testing include over-diagnosis of food allergy, overly restrictive diets, malnutrition, the risk of conversion to anaphylactic food allergy after a period of avoidance of previously tolerated foods (see statement #22), and unnecessary costs to the health care system for unwarranted testing.^124^ These unnecessary costs may even extend to physician supervised oral food challenge procedures for re-introduction of panel-tested allergenic foods, to rule out iatrogenic conversion from sensitization to true IgE-mediated food allergy.

**Statement #30: In addition to shared understanding and experience in the pathophysiology and management of EoE with gastroenterologists, allergists have unique experience in their ability to manage concurrent and complicating allergic conditions, such as IgE-mediated food allergy and determination of relevant aeroallergen sensitization.**

Multidisciplinary EoE care in many academic and community clinics involves collaboration between gastroenterologists and allergists, due to shared understanding and experience in the pathophysiology and management of EoE.^65^ In some centres, allergists may act as the principal provider of care for patients with EoE, in collaboration with a gastroenterologist/endoscopist (see statement #9 and Canadian context section).^44^ Allergists have unique training and experience in diagnosis and management of concurrent allergic conditions, including atopic dermatitis, anaphylactic IgE-mediated food allergy, asthma, and allergic rhinitis, with most allergists routinely prescribing medications such as antihistamines, topical/inhaled/intranasal corticosteroids, and biologics.^1^^,^^133^ Allergists can perform diagnostic oral food challenges for EoE patients on restrictive diets, when there has been a history of possible immediate reaction upon accidental exposure and/or positive skin prick/sIgE testing.^133^ Allergists can test for sensitization to aeroallergens and possible exacerbation of EoE due to aeroallergen exposure.^133^ Allergists can help EoE patients weigh the benefits/risks of treatment with sublingual (aeroallergen or food) and/or oral (food) immunotherapy.^134^ Biologics (see statement #26) to treat EoE may be prescribed by either a gastroenterologist or allergist, depending on local resource limitations and access. For example, biologics to treat EoE may be prescribed by allergists in centres when they are the principal providers of care, there is a lack of access to gastroenterologists, or if the patient has multiple allergic conditions.^135^

**Statement #31: A subset of patients sensitized to pollen may experience seasonal intensification of EoE due to pollen allergy. Allergists can help patients distinguish between EoE and Pollen Food Allergy Syndrome to better manage symptoms.**

A subset of EoE patients sensitized to pollen may experience seasonal exacerbation of EoE.^136^ Proposed mechanisms include swallowing pollen that has been inhaled (resulting in direct contact with esophageal mucosa), or release of inflammatory mediators (from the airway of patients with allergic rhinitis or asthma) that circulate via the bloodstream to the esophagus.^137^ The extent of this association is unclear and warrants further research, with a 2015 systematic review of 16,846 EoE patients not supporting a clear causal role for aeroallergens.^138^ Among a recent retrospective cohort of 782 EoE patients in North Carolina, 13 (2%) patients met strict criteria for seasonal EoE exacerbation. This was defined as patients with histologic remission (<15 eos/hpf) outside of the pollen seasons who at least doubled their esophageal eosinophil counts during relevant pollen seasons without change in EoE therapy.^139^ The study suggested that this is an uncommon association, although the stringent definition of flare may have underestimated the association, especially in patients who have not yet achieved histologic remission. Beyond identifying this association in a subset of EoE patients, there is very little literature documenting improvement or remission of EoE via aeroallergen avoidance, medical management of allergic rhinitis and/or asthma, or aeroallergen immunotherapy.^140^ In EoE patients who are sensitized to pollen, allergists can help distinguish between seasonal exacerbation of EoE due to pollen exposure versus pollen food allergy syndrome (PFAS). PFAS is highly associated with EoE, with 26% of US cohort having both conditions concurrently.^141^ In contrast with EoE, pollen-food allergy syndrome (PFAS) most commonly manifests as oropharyngeal symptoms (eg, oropharyngeal pruritus) upon exposure to foods such as raw fruits/vegetables that cross-react with pollen, with anaphylactic symptoms being rare.^142^ Being able to tolerate processed (eg, cooked) forms of these foods is highly suggestive of PFAS, as processing denatures the highly labile proteins responsible for eliciting the symptoms. Although EoE and PFAS can coexist, the disorders can be clinically distinguished because PFAS does not directly cause chronic dysphagia and food impaction; PFAS symptoms will be present immediately after exposure and will be limited mainly to the oropharynx.

**Statement #32: It is unclear whether sublingual or oral immunotherapy causes or unmasks EoE, or whether the disease is simply associated with the therapy. Immunotherapy should be based on weighing benefits/risks and shared decision-making.**

A systematic review with meta-analysis found overall prevalence of EoE after oral immunotherapy (OIT) to be 2.7% (95% CI, 1.7%-4.0%, *I*^2^ = 0%), with EoE often resolving after OIT discontinuation.^143^ More recent OIT clinical trials and real world OIT data have described a lower risk of EoE during OIT (<1%).^144^^,^^145^ A possible mechanism for EoE that develops during OIT is repeated antigen stimulation from OIT driving differentiation of T_H_2 cells into peT_H_2 cells (pathogenic effector T_H_2 cells) expressing IL-5.^146^ In the research setting, it appears EoE induced by OIT may be transient (onset during the OIT buildup phase with resolution during the OIT maintenance phase) and not always associated with GI symptoms.^147^ In the clinical setting, it is unclear whether sublingual (aeroallergen or food) or oral (food) immunotherapy causes or unmasks EoE, or if their timing is coincidental. Patients undergoing these forms of immunotherapy outside of research setting do not undergo baseline endoscopy and biopsies prior to immunotherapy, making it impossible to rule out pre-existing esophageal eosinophilia. In a prospective cohort of 89 patients with persistent anaphylactic cow’s milk allergy, 38.2% (95% CI, 28.14%-49.16%) had esophageal eosinophilia defined as >15 eos/hpf with many being asymptomatic or having non-specific symptoms, leading the authors to suggest that EoE can be “silent” in patients with IgE-mediated food allergy even without OIT.^148^ Due to this confusion about causation, allergists have traditionally been apprehensive to continue sublingual or oral immunotherapy once EoE has been confirmed, with, eg, licensed OIT and SLIT (sublingual immunotherapy) product monographs listing EoE as a contraindication to continuing OIT or SLIT.^146^ With time, however, it appears the decision to continue SLIT or OIT (oral immunotherapy) should be anchored in weighing benefits/risks and shared decision-making, similar to whether a patient should continue SLIT or OIT in the context of other concomitant allergic conditions.^134^ Indeed, the recent Canadian OIT guidelines list EoE as a relative contraindication rather than an absolute contraindication, recommending that the decision should be based on clinical judgement, provider expertise, and shared decision-making.^149^ Real world data has demonstrated that OIT can be successfully maintained in most patients with use of interventions such as PPI or swallowed topical corticosteroids to control EoE, when family and patient preference is considered.^150^^,^^151^ A shared decision-making practical guide for managing GI symptoms during OIT was recently published and has a suggested flow diagram (Figure S1).^152^

**Statement #33: When feasible, single medical interventions or monotherapy (Diet/medications) should be evaluated in isolation to know their effectiveness. Combination therapies may be considered for more severe disease.**

Through the patient’s journey, therapeutic trials are necessary. For patient simplicity and for the practitioner’s benefit, evaluating a single intervention is ideal to allow development of an understanding of the therapeutic benefit of each intervention.^153^ Pragmatically, the selection of single, or multiple simultaneous approaches, will be driven by patient and practitioner factors. The benefit of evaluating single intervention changes, is that precise identification of the intervention effect is clearer. While there are possibilities of partial responses or synergistic benefit of more than 1 therapy, for example a combination of a milk free diet and PPI,^154^ adherence to a single therapy is likely to be better. Circumstances where the benefit of a single intervention approach may not be as clear include when a patient has severe symptoms and corresponding stricture. In this case, treatment with medications/diet in addition to dilation may be the best approach.^155^

**Statement #34: Clinical evaluation alone is not sufficient to assess treatment efficacy. Repeat endoscopy and biopsy to assess treatment efficacy after a change in management ideally should occur at 6-12 weeks.**

While symptomatic improvement and relief is of primary interest to the patient, there is also value in evaluating histologic improvement (plus or minus endoscopic) with a prescribed therapy as an objective marker. The need for monitoring more than symptom improvement is a reflection of the poor correlation between histology and symptoms.^156^^,^^157^ In addition, patients often have very chronic symptoms and frequently have developed adaptive behaviours which may minimize symptoms. Therefore, evaluating aspects other than symptoms is important. Histological and/or endoscopic monitoring will allow practitioners to support decisions about whether a therapy should be continued, modified or changed completely and monitor for complications.^158^ Recent meta analysis of clinical trial designs in EoE show symptom response to placebo is high at 40%, whereas histologic and endoscopic response is very low.^159^ There are presently no readily available biomarkers which allows for evaluating disease status. Tests such as EndoFLIP (Medtronic, United States) may play a role in describing esophageal distensibility and wall stiffness, and provide complimentary information about esophageal function^32^^,^^33^ but this is not widely available. As of now, the eosinophil count from biopsies remains the gold standard to represent remission with current adoption of ≤15 eos/hpf as the most common cutoff, and more stringent cutoff (<6 eos/hpf) used for clinical trials.^160^

**Statement #35: It is important to ensure follow-up of EoE. The interval for follow up including endoscopy may vary depending on symptoms (frequency and severity), phenotype including history of strictures, and amount of inflammation, with uncontrolled EoE requiring more frequent follow-up.**

The ideal time interval between clinic review or even endoscopy is hard to prescribe without factoring in individual patient information such as access to endoscopy, current symptoms, duration of treatment trial and history of strictures to name a few. As such, episodic care, or on-demand care is not ideal.

Routine clinic visits can allow for symptom review, both frequency and severity, to assess medication side effects, barriers to treatment, anthropometric review, understanding of patient treatment options (including new treatments) and ensuring patient values are in line with current treatment.^158^^,^^161^ Episodic and on demand care does not allow for this evaluation, or the development of a therapeutic relationship, which would improve patient adherence to treatment and collaborations amongst various stakeholders.

**Statement #36: Disease activity indices for symptomatic, endoscopic, histologic, and quality-of-life measures are available and can be used, although may not always be feasible to adopt in routine clinical settings.**

The definition of severe disease is not universal amongst patients or clinicians. While most would agree the presence of a stricture or a food bolus impaction defines a more severe phenotype, this is not predictive of which therapies may or may not work.

A holistic perspective is required to ensure improvement from multiple angles.^162^ Validated symptom questionnaires are available including DSQ (Dysphagia Symptom Questionnaire),^163^ PEESS2.0^164^ and more. While these allow for a consistent approach to assessing patient symptoms, application of these scales is cumbersome and challenging in clinical care. At this time, there is no one perfect score. I-SEE was developed to incorporate multiple angles of patient dysfunction, such as symptoms (frequency), complications, endoscopic features (inflammatory and fibrostenotic) (see statement #11) and histologic features (eos counts, lamina propria fibrosis, etc). Putting all these into a composite score, it allows the physician to stratify disease into mild, moderate and severe types.^165^^,^^166^ Another aspect of EoE that is important to capture is quality of life. PedsQL EoE module^167^ has been developed and validated for children/adolescents based on the validated EoE QOL questionnaire used in adults.^168^

**Statement #37: EoE has a negative impact on psychosocial status. Monitoring the overall well-being of EoE patients is an important part of follow-up.**

Eosinophilic Esophagitis affects patients in numerous different ways. Chronic diseases affect psychosocial wellbeing and typically are associated with a lower QOL. Studies show QOL with EoE is negatively impacted in both adults and pediatrics. It is important to keep in mind QOL can be negatively affected even in those with the disease being under control. Factors that play into this include the need for follow up with repeat endoscopy, and therapeutic challenges (be it from a focus on what they cannot eat, the meds they need to take, the time they are told not to eat after taking a med, the administration of medication, the costs associated with treatment, and/or worrying about potential negative outcomes). Feeding aversions can develop and a resultant poor relationship with food, including ARFID.^169^ All this can play into the disease specific negative psychological status or contribute to development of mental health conditions including anxiety and depression.^170–172^

In a retrospective review of psychological evaluations of EoE children, 64 psychological evaluations were reviewed including disease-related pain/discomfort; feeding/appetite symptoms; sleep, social, and school problems; depression, anxiety; and overall psychological adjustment: 69% experienced some form of psychosocial issue; 64% experienced social difficulties; 41% anxiety; 33% sleep difficulties; 28% depression; and 26% school problems; 44% had adjustment problems—this was mainly observed in older children. For older children adjustment issues were predominantly school related, social difficulties, anxiety and depression. For younger (0-4), it was somatic and emotional regulation, sleep disturbances, and feeding problems.^173^ The impact of EoE on caregivers is poorly understood. Therefore, understanding and monitoring psychosocial health of patients and families is critical for EoE patient care.

**Statement #38: The ultimate duration of therapy for patients who achieve control of their EoE is unclear in the literature. Given this is a long-term condition, the decision to continue treatment and in what form is dependent on severity of symptoms and disease, as well as shared decision-making with the patients and family, and balancing risk and benefits of the treatment with the risks of complications (eg, fibrostenotic disease).**

It is important to consider that relapse of EoE is frequent, especially with cessation of treatment. Therefore, there is a need for maintenance strategies. However, there are no clear evidence-based recommendations regarding long-term follow-up and treatment.^161^ Recent publications from various countries are unanimous in suggesting long-term treatment should continue with the agent that induced remission of EoE. However, data is limited to determine efficacy of this approach and which patient population is best served by this path. What we do know is that lack of treatment certainly results in recurrence of inflammation and rising rates of strictures and food impactions. If patients find benefit with a specific medication, proposed management options include switching to lower dose or continuing with same dose indefinitely. However, with long-lasting remission, the benefits and risks of treatment discontinuation versus prolongation need to be discussed with the patient. There is not enough data to support or reject the discontinuation of treatment when long-term remission is apparent.^174^

### Canadian context, advocacy, and future research

This study presents a comprehensive set of recommendations for EoE care in Canada that incorporates both expert opinion and evidence. Strengths of this approach include the diverse and multidisciplinary group of experts included that represented health regions across Canada and the many specialties that are required for effective EoE care. Although we aimed to include a representative expert panel and ensured consensus through the iterative nature of the recommendation development process, we must acknowledge that each expert brings their own biases and focus and it is unclear how this may have influenced the final statement.

Finally, in developing this guideline we note that the diagnosis and management of EoE within the Canadian healthcare system is unique. We encourage EoE centres in Canada to come together in a multidisciplinary format to not only provide clinical care but also do much needed research on Canadian specific topics and gaps in EoE care. Specific areas of future research highlighted throughout evidence summaries as part of the EoE recommendations and are summarized in Table 2. Finally, to best support patients with EoE, advocacy for prescription drug coverage for all described treatments is needed to ensure the best outcomes for our patients in an equitable fashion.

**Table 2.** Gaps and future research areas needed to further support evidence-based care of EoE across age groups in Canada and globally.

	Statement	Research need/gap
**Section 1: Definition**	1	Further research to better understand pathophysiology of EoE to support development of effective treatments and management strategies. This includes better prognosis and severity indexes to support improved patient outcomes.
	2 & 3	Epidemiological studies in Canadian populations to understand current burden of care and reasons for increasing diagnosis. Additional focus of data collection on burden of disease in Black, Hispanic, and Indigenous populations is also needed, particularly in the setting of type 2 allergic conditions such as asthma, IgE-mediated food allergy, atopic dermatitis, and allergic rhinitis.
**Section 2: Diagnosis**	9	Research to further understand impact of rural location on rates of EoE and if there is a true association between the two.
	11 & 14	Prospective, clinical validation of I-SEE or a similar prognostic score is required to guide clinical assessments of severity. Validation of EoEHSS or a simpler tool for clinical practice that is easily applicable and clinically relevant for practice
	12	A component of this work should include evaluating different biopsy regimens including number of biopsies per pass. In addition, research to determine the most effective method of biopsy collection to support diagnosis. Continued research is needed on innovative methods for diagnosing and following EoE including noninvasive biomarkers or less invasive testing which can be feasibly implemented in a clinical setting.
**Section 3: Management**	16, 20, & 30	Evaluate and facilitate the development of teams and encourage data sharing across clinics to enhance sample size and strength of analysis for all further EoE research.
	17	Development and evaluation of programs that support transition of care in the Canadian context. These programs should facilitate better patient education, treatment adherence, and sharing of health information.
	22	Understand the risk and relationship between EoE and the development of severe IgE-mediated food allergies after an elimination diet is needed to more safely guide use of this management technique.
	25	Gather and analyze real-life effectiveness data on the off-label use of swallowed topical corticosteroids beyond short term including dose variation for maintenance use.
	26	Support development of treatment strategies that are novel. Priority should be given to development of treatments that are effective, easy to administer from the patient perspective and of lower cost.
	27	Policy research to support universal EoE drug coverage in Canada, based on Canadian cost-effectiveness analyses.
	29	Development of investigations that are predictive of food triggers of EoE.
	31	Causal studies to clarify the relationship between pollen sensitization and seasonal EoE variations.
	32	Causal studies to understand the relationship between OIT and EoE to clarify the pathophysiological mechanisms.
	33, 35, & 36	Develop evidence to support management pathways and better understand when monotherapy is required versus combined therapies. Similarly, time interval of monitoring with clear guidance on follow-up endoscopy interval based on patient condition and prognosis remains unclear. Prognostic models that can be applied to individual patients to guide care and monitoring.
	34	Research to determine feasibility and utility of measuring distensibility using endoFLIP (Medtronic, United States) in clinical care prior to cost-effectiveness analysis.^a^
	37	Research to understand impact of EoE on caregivers and support development of effective management strategies that incorporate family support.
	38	Data on natural history, long-term follow-up, and treatment use to guide management decisions.

a EoE = Eosinophilic Esophagitis; I-SEE = Index of Severity for Eosinophilic Esophagitis; EoEHSS = Eosinophilic Esophagitis Histologic Scoring System; IgE = Immunoglobulin E; OIT = Oral Immunotherapy; endoFLIP = endoluminal functional lumen imaging probe;

## Supplementary Material

gwaf022_Supplementary_Data

## Data Availability

The survey data underlying this article will be shared on reasonable request to the corresponding author.
