# Supplementary material for: Recommendations for the diagnosis and management of eosinophilic esophagitis in adults and children in Canada: a Delphi consensus project
Source: J Can Assoc Gastroenterol. 2025 Nov 6;8(6):207–27. doi: 10.1093/jcag/gwaf022 (PMC12698229; doi:10.1093/jcag/gwaf022)
Supplement: gwaf022_Supplementary_Data [file gwaf022_supplementary_data.zip › DelphiEoE_Supp_20250401_revised.docx]

**Supplementary Appendix 1**

**Detailed Methods**

This study was a modified Delphi consensus project and was not registered. The Delphi technique is characterized by a series of rounds that ask for the opinions of experts on a particular topic. This is an evolving process as each round builds on findings from the previous round resulting in a final consensus on the topic of interest. This method is often used when developing tools, guidelines, competencies, anything that requires the knowledge and experience of experts within a field where supportive literature may be limited^1^.

*Study participants*

The study steering committee (EC, VA, MG, CM) identified a national multidisciplinary expert group of 31 specialists through personal networks and snowball sampling to participate in the Delphi consensus process. Inclusion in the expert group required members to be currently practicing clinically and have experience with EoE care in Canada within one of the following professional groups: allergist, adult gastroenterologists, pathologists, pediatric gastroenterologists, and dieticians. Experts were chosen to reflect a variety of experiences and geographic locations to ensure diverse representation from all across Canada (Supplementary Table 1). Each potential expert member was invited to participate through direct email by the study coordinator and provided an informed consent form to review and sign if they agreed to participate. Steering Committee members also participated as voting members in the expert group throughout the Delphi process.

*Delphi procedures*

A preliminary list of recommendations was developed by the study steering committee through a combination of literature search and expert knowledge. The literature search was performed by study team member HA using the MeSH term: eosinophilic esophagitis. The search was further refined and repeated using each of the truncated terms diagnose*, treat*, and manage*. Studies were filtered to include reviews, meta-analyses, clinical trials, control trials, and observational studies. Only articles written in English were selected. Resulting articles were included for review if they discussed topics related to EoE epidemiology, diagnosis, management or treatment of any type that occurred in Canada, and/or involved randomization and/or were systematic reviews with or without meta-synthesis. References from included articles were also reviewed to identify any articles potentially missed in the original search. A final search was conducted to identify all comparable EoE Clinical Practice Guidelines from other jurisdictions. Evidence identified in the literature search was summarized as an annotated bibliography and provided to each Steering Committee member to review prior to drafting the initial statement set. The annotated bibliography was also shared with expert group members once the Delphi rounds began and all authors were given opportunity to add the this list. Once the initial set of statements was defined, the statements were then divided into three sections: definition, diagnosis, and management.

For each round of Delphi survey, expert group members including the steering committee members rated their agreement with each statement on a Likert scale of 1 to 9; 1-3 indicating disagreement, 4-6 indicating neutrality and 7- 9 indicating agreement. At the end of each section, they were also given the opportunity to suggest edits to the statements as well as suggest new statements. Expert group members were not required to explain their ratings directly on the survey and were not given incentives to participate. Surveys were completed individually and anonymously using REDCap (Research Electronic Data Capture; Vanderbilt University). The survey tool was not piloted prior to use and was available in English only as no expert group member required adaptation to participate.

Scaled survey responses were analyzed after each survey round using descriptive statistics. Analysis included the median, interquartile range, mode, absolute range, percent agreement (number of votes >=7), and Fleiss Kappa coefficient. All analyses were conducted using R Studio (). The interpretation of the Kappa was based on standard criteria as follows: κ value < 0.00 indicates poor agreement, 0.00–0.20 slight, 0.20 to 0.40 moderate, 0.61 to 0.80 substantial and > 0.81 almost perfect agreement^2^. Consensus was defined based on two primary quantitative criteria: (1) at least 80% agreement (rating of 7 or above) on the Likert scale responses; and (2) a Kappa score >0.61.

After each survey, an anonymized aggregate summary of quantitative results and participant comments was circulated to the expert group for review. Statements that reached agreement based on consensus criteria for any given survey and did not have any comments were approved for inclusion in the final list of recommendations and not included in any subsequent Delphi rounds. Statements that did not reach consensus criteria in the survey were discussed and wording edited in a 90-minute virtual Zoom consensus meeting. A virtual whiteboard was created to allow collaboration on re-wording of the statements during the consensus meeting. Participants had to complete each survey in order to attend the consensus meeting and proceed to the following round of Delphi; however, a participant did not have to attend the consensus meeting to move on to the next round of Delphi and be invited to rate statements on the next survey.

Based on the group discussion, any statement that did not previously meet the consensus criteria was included in the next survey to be re-rated by the expert group using the Likert scale. Due to time constraints, not all statements could be discussed at each consensus meeting. When this occurred, any remaining statements were reviewed and edited by the SC for inclusion in the subsequent survey. This process was planned to be repeated for a minimum of three Delphi rounds. For the final round of consensus only, statements that did not meet both criteria for agreement in the survey were discussed, edited and re-rated for agreement within the meeting itself. Any statements that did not reach agreement at this point were planned to be excluded from the final list of recommendations.

*Dissemination and implementation of guidelines*

The aim of this guideline is to provide recommendations for care that can be implemented within the current constraints and reality of the Canadian healthcare system. Where possible, acknowledgement of current limitations in our system has been incorporated to contextualize the recommendations and provide room for health system improvement. These guidelines will be disseminated through publication and presentation and various academic and clinical practice meetings across the country. We expect to update these guidelines every # years to ensure they stay valid and representative of Canadian patient and care provider needs.

References

1. Barrett D, Heale R. What are Delphi studies? *Evidence-Based Nursing*2020;**23:**68-69.
2. Landis, J. R., & Koch, G. G. (1977). The Measurement of Observer Agreement for Categorical Data. *Biometrics*, *33*(1), 159–174. https://doi.org/10.2307/2529310

Supplementary Table 1: Characteristics of the Delphi expert group and participation rates in each Delphi round

#

| **Characteristic** | **Total**  **n(%)** | **Round 1 survey** | **Round 1 meeting** | **Round 2 survey** | **Round 2 meeting** | **Round 3 survey** | **Round 3 meeting** |
| --- | --- | --- | --- | --- | --- | --- | --- |
| Gender |  |  |  |  |  |  |  |
| *Male* | 18 | 18 | 18 | 18 | 15 | 18 | 14 |
| *Female* | 13 | 13 | 12 | 13 | 11 | 13 | 11 |
| Geographic region of clinical practice in Canada |  |  |  |  |  |  |  |
| *Western provinces* | 14 | 14 | 14 | 14 | 11 | 14 | 11 |
| *Central* | 14 | 14 | 13 | 14 | 12 | 14 | 12 |
| *Eastern* | 3 | 3 | 3 | 3 | 3 | 3 | 3 |
| Clinical specialty |  |  |  |  |  |  |  |
| *Adult GI* | 9 | 9 | 9 | 9 | 7 | 9 | 7 |
| *Peds GI* | 9 | 9 | 8 | 9 | 8 | 9 | 7 |
| *Allergists* | 9 | 9 | 9 | 9 | 8 | 9 | 8 |
| *Dietician* | 2 | 2 | 2 | 2 | 2 | 2 | 2 |
| *Pathologist* | 2 | 2 | 2 | 2 | 1 | 2 | 2 |

# Results Supplement:

# In round 1, 22 statements met the consensus threshold after the first survey. At the first consensus meeting, 26 statements were discussed, including two new statement suggestions. All discussed statements were included in survey 2 for agreement rating. In Delphi round 2, 15 additional statements met the consensus threshold. There were 11 statements discussed in the virtual consensus meeting and included in survey 3. As a result of the second consensus meeting, 2 statements in the management section that dealt with timing of endoscopic follow-up were combined for ease of understanding and in order to be more concise. In round 3, a final seven statements met the consensus threshold while four were discussed in the virtual consensus meeting and voted on at a final time during the meeting. After round 3, statements in all sections were re-ordered to ensure the flow of recommendations followed an expected clinical process.

# For the following 3 tables, a total of 31 experts responded to the Delphi Survey in each round. Consensus is determined based on (1) 80% or above agreement defined by number of responses at or above 7 on the 9-point likert scale for statement agreement; and (2) a Kappa score >0.61. Any statement that met BOTH consensus criteria in this survey is highlighted green. Statements that met only ONE consensus criteria are highlighted yellow and statements that have met NONE of the consensus criteria are highlighted orange. In subsequent tables statements that previously met consensus criteria are highlighted blue. In the consensus meeting all yellow and orange highlighted statements and new statement suggestions were discussed and reworded.

**Supplementary Table 2: Aggregate Delphi Round 1 survey results.**

| Recommendation | Median ranking | IQR | Mode | Absolute Range | n>=7 | Kappa |
| --- | --- | --- | --- | --- | --- | --- |
| DEFINITION |  |  |  |  |  |  |
| **Statement #1: EoE provider care should be a collaborative effort between multiple providers (gastroenterologists, allergists, surgeons, pathologists, dietitians, and primary care practitioners).** | 9 | 9-9 (0) | 9 | 5-9 | 28 (90.3%) | 0.734 |
| **Statement #2: EoE is a chronic allergic inflammatory condition of the oesophagus, involving barrier dysfunction, type 2 inflammation, eosinophilic infiltration, and tissue remodelling.** | 9 | 9-9 (0) | 9 | 7-9 | 31 (100%) | 0.869 |
| **Statement #3: EoE is increasing in prevalence, which can be partially attributed to increased awareness. The true rate is not well defined in Canada.** | 9 | 8-9 (1) | 9 | 5-9 | 29 (93.5%) | 0.710 |
| **Statement #4: EoE predominantly affects males and Caucasians, and is highly associated with atopic conditions such as IgE-mediated food allergy, asthma, atopic dermatitis, and allergic rhinitis.** | 8 | 8-9 (1) | 9 | 4-9 | 27 (87.1%) | 0.563 |
| **Statement #5: Long term complications of EoE include fibrosis and stricture development, with increased risk from diagnostic delay and untreated EoE.** | 9 | 8-9 (1) | 9 | 7-9 | 31 (100%) | 0.769 |
| **New statements suggested** |  |  |  |  |  |  |
| DIAGNOSIS |  |  |  |  |  |  |
| **Statement #6: EoE is defined clinically by symptoms of esophageal dysfunction together with esophageal biopsy showing ≥15 eosinophils/hpf.** | 9 | 8-9 (1) | 9 | 3-9 | 28 (90.3%) | 0.614 |
| **Statement #7: EoE symptoms and signs in younger children include feeding difficulties, abdominal pain, failure to thrive, and vomiting.** | 9 | 8-9 (1) | 9 | 5-9 | 29 (93.5%) | 0.714 |
| **Statement #8: EoE symptoms in older children, adolescents and adults include dysphagia and food impaction.** | 9 | 8.5-9 (0.5) | 9 | 5-9 | 29 (93.5%) | 0.742 |
| **Statement #9: Given waiting lists for GI in Canada can be long, patients with possible EoE may be trialled on high-dose PPI for symptomatic relief while waiting for consultation and endoscopy. Severe symptoms including food impaction require immediate referral to GI.** | 8 | 7-9 (2) | 9 | 2-9 | 24 (77.4%) | 0.296 |
| **Statement #10: In situations where access to GI is prolonged or unavailable, for example in rural and remote regions, general surgeons may provide initial access to endoscopy and biopsy for EoE diagnosis and then collaborate with medical practitioners for management.** | 8 | 7-8.5 (1.5) | 9 | 1-9 | 24 (77.4%) | 0.405 |
| **Statement #11: A trial of proton pump inhibitors is not required for diagnosis of EoE.** | 9 | 8-9 (1) | 9 | 5-9 | 29 (93.5%) | 0.711 |
| **Statement #12: Biopsies from upper GI endoscopy are mandatory for diagnosis. There should be ≥2 biopsies per level from at least two levels (distal, mid, and proximal esophagus), for a total of ≥6 biopsies. Biopsies of the stomach and duodenum are also recommended to rule out eosinophilic gastritis and duodenitis.** | 9 | 7.5-9 (1.5) | 9 | 5-9 | 28 (90.3%) | 0.610 |
| **Statement #13: Gross endoscopic findings for EoE can include edema, rings, exudates, linear furrows, and stricture. They should be reported as EREFS including subscores. One can have increased eosinophils without macroscopic features.** | 9 | 8-9 (1) | 9 | 7-9 | 31 (100%) | 0.746 |
| **Statement #14: In the event of a food impaction, if endoscopy is performed, biopsies should also be taken so that proper diagnosis and management can follow urgently. High-dose PPI is reasonable at this time.** | 9 | 8-9 (1) | 9 | 6-9 | 29 (93.5%) | 0.689 |
| **Statement #15: Histology should be reported as eosinophil counts per 0.3mm2 (=per hpf), with EoE diagnosis requiring ≥15 eosinophils/hpf.** | 9 | 9-9 (0) | 9 | 7-9 | 31 (100%) | 0.856 |
| **Statement #16: Classic features beyond esophageal eosinophlia can include dilated intercellular spaces, basal zone hyperplasia, eosinophilic abscesses as well as lamina propria fibrosis, and can complement a diagnosis.** | 9 | 8-9 (1) | 9 | 7-9 | 31 (100%) | 0.749 |
| **Statement #17: The differential diagnosis of EoE includes gastroesophageal reflux disease (GERD), eosinophilic gastrointestinal disease (EGID), Crohn's disease, achalasia, hypermobility syndromes, drug hypersensitivity disorder, and others.** | 9 | 8-9 (1) | 9 | 6-9 | 30 (96.8%) | 0.695 |
| **New Statements** |  |  |  |  |  |  |
| MANAGEMENT |  |  |  |  |  |  |
| **Statement #18: It is important to transition care from paediatric to adult services to support them through this high risk period.** | 9 | 8.5-9 (0.5) | 9 | 5-9 | 29 (93.5%) | 0.704 |
| **Statement #19: Initial management options for controlling symptoms, reducing inflammation, and preventing complications include PPI, dietary elimination, and swallowed topical corticosteroids. For severe disease, dilation and biologics are further options.** | 9 | 8-9 (1) | 9 | 2-9 | 27 (87.1%) | 0.535 |
| **Statement #20: Although repeat endoscopy and biopsy can occur as soon as 8 weeks after a change in management, in most Canadian healthcare settings 3-4 months is more feasible.** | 8 | 7-9 (2) | 9 | 1-9 | 24 (77.4%) | 0.379 |
| **Statement #21: Mode of medication delivery (pill, slurry, etc.) is a shared decision-making process between patient and provider to ensure compliance.** | 9 | 8-9 (1) | 9 | 5-9 | 30 (96.8%) | 0.660 |
| **Statement #22: Empiric dietary elimination is best started with one (cow’s milk) or two (cow’s milk and wheat) food elimination, balancing efficacy, convenience, and adherence. A six-food elimination diet is less convenient, and more difficult to adhere to.** | 8 | 8-9 (1) | 9 | 2-9 | 27 (87.1%) | 0.522 |
| **Statement #23: Consultation with a dietitian is recommended for patients on dietary elimination.** | 9 | 8-9 (1) | 9 | 7-9 | 31 (100%) | 0.740 |
| **Statement #24: An elemental diet is rarely required for management of EoE, due to lack of oromotor skill development when used in young children, poor taste, and high cost.** | 8 | 8-9 (1) | 9 | 6-9 | 27 (87.1%) | 0.643 |
| **Statement #25: Atopic patients who have undergone any form of prolonged dietary elimination may be at higher risk of conversion to IgE-mediated food allergy to the foods that have been avoided.** | 8 | 7-9 (2) | 9 | 5-9 | 24 (77.4%) | 0.475 |
| **Statement #26: Budesonide orodispersible tablet is approved by Health Canada for the treatment of EoE. Off label swallowed topical corticosteroid options include viscous budesonide and fluticasone MDI. However, the hydrofluorocarbon propellants in MDIs are major greenhouse gases and contribute to climate change.** | 7 | 5.5-8.5 (3) | 9 | 2-9 | 21 (67.7%) | 0.225 |
| **Statement #27: Systemic (e.g. oral prednisone) corticosteroids are not recommended for treatment of EoE.** | 8 | 7-9 (2) | 9 | 2-9 | 24 (77.4%) | 0.374 |
| **Statement #28: Stricture development can be a complication of the disease. While endoscopic dilation may alleviate symptoms it is important to address stricture through antiinflammatory treatments such as topical corticosteroids.** | 8 | 8-9 (1) | 9 | 4-9 | 29 (93.5%) | 0.611 |
| **Statement #29: Dupilumab is the only Health Canada approved biologic for EoE. Anti-IL-5 monoclonal antibodies have shown histologic improvement but persistence of symptoms in clinical trials. There are other biologics currently being evaluated in EoE clinical trials.** | 9 | 7-9 (2) | 9 | 1-9 | 26 (83.9%) | 0.419 |
| **Statement #30: The cost of Health Canada-licensed EoE medications can be high. Due to variable coverage by 3rd party prescription programs and lack of current coverage by provincial prescription drug plans, a coordinated effort is needed to ensure patients with EoE receive appropriate treatments regardless of location.** | 9 | 9-9 (0) | 9 | 7-9 | 31 (100%) | 0.830 |
| **Statement #31: Allergy testing (skin prick tests, sIgE blood tests, or patch tests) to uncover food triggers of EoE is not recommended due to poor predictive value. Rather, if chosen dietary elimination should be done empirically. The purpose of food allergy testing is to rule out potentially anaphylactic IgE-mediated food allergy when the history is suggestive of it.** | 9 | 8-9 (1) | 9 | 6-9 | 30 (96.8%) | 0.740 |
| **Statement #32: Allergists play an important role in multidisciplinary EoE management, such as selection and interpretation of skin prick or sIgE tests prior to re-introduction of previously eliminated foods, oral food challenges to rule out conversion to IgE-mediated food allergy, management of associated atopic conditions (food allergy, asthma, atopic dermatitis, allergic rhinitis), and conducting allergen immunotherapy (e.g. environmental or food immunotherapy).** | 9 | 8-9 (1) | 9 | 2-9 | 29 (93.5%) | 0.579 |
| **Statement #33: A subset of patients sensitized to pollen may experience seasonal intensification of EoE due to pollen allergy. Allergists can help distinguish between EoE and Pollen Food Syndrome.** | 8 | 7-9 (2) | 9 | 5-9 | 26 (83.9%) | 0.514 |
| **Statement #34: Initiation or continuation of allergen immunotherapy (e.g. sublingual or oral) in the setting of EoE is an option and should be based on weighing benefits/risks and shared decision-making, due to possible worsening of EoE. It is unclear whether sublingual or oral immunotherapy causes or unmasks EoE, or whether it is coincidental.** | 8 | 7-9 (2) | 9 | 2-9 | 24 (77.4%) | 0.308 |
| **Statement #35: Recommended follow-up for uncontrolled EoE is every 3-4 months, versus 12-24 months for well-controlled EoE.** | 8 | 8-9 (1) | 9 | 1-9 | 27 (87.1%) | 0.500 |
| **Statement #36: Severity indexes and quality-of-life instruments should be adopted and further developed so they are feasible for use in the clinical setting.** | 8 | 7-9 (2) | 9 | 2-9 | 26 (83.9%) | 0.467 |
| **Statement #37: Symptoms alone are inadequate to follow the disease state. Adherence with treatment should be reviewed at follow-up, in addition to endoscopic surveillance.** | 9 | 8-9 (1) | 9 | 2-9 | 28 (90.3%) | 0.489 |
| **Statement #38: EoE has a negative impact on psychosocial status. Monitoring the overall well-being of EoE patients is an important part of follow-up.** | 9 | 8-9 (1) | 9 | 6-9 | 30 (96.8%) | 0.731 |
| **Statement #39: The ultimate duration of therapy for patients who achieve control of their EoE is unclear in the literature. Given this is a long-term condition, if therapy is stopped there will be risks such as disease recurrence or stricture development.** | 9 | 8-9 (1) | 9 | 5-9 | 28 (90.3%) | 0.653 |
| **New statements** |  |  |  |  |  |  |

# Supplementary Table 3: Aggregate Survey round 2 results

| Recommendation | Median ranking | IQR | Mode | Absolute Range | n>=7 | Kappa |
| --- | --- | --- | --- | --- | --- | --- |
| DEFINITION |  |  |  |  |  |  |
| **Statement #1: EoE is a chronic inflammatory condition of the esophagus, represented by eosinophilic inflammation. Some of the pathophysiology that contributes to this condition includes barrier dysfunction, delayed (non-IgE) allergic responses to food (and or environmental allergens), type 2 inflammation and tissue remodelling. Rate your level of agreement with this statement.** | 9 | 8-9(1) | 9 | 7-9 | 31  (100%) | 0.792 |
| **Statement #2: EoE is increasing in prevalence, which can be partially attributed to increased awareness. The true prevalence is not well defined in Canada.** |  |  |  |  |  |  |
| **Statement #3: EoE can be seen in all genders and ethnic backgrounds but is more commonly diagnosed in males and people of white race. It is often associated with allergic conditions such as IgE-mediated food allergy, asthma, atopic dermatitis, and allergic rhinitis. EoE can be associated with family history of EoE in first degree relatives, especially those with allergies.** | 9 | 7-9(2) | 9 | 5-9 | 28 (90.3%) | 0.579 |
| **Statement #4: Long term complications of EoE include fibrosis and stricture development. These risks are increased with diagnostic delay and untreated EoE.** |  |  |  |  |  |  |
| **New statements suggested** |  |  |  |  |  |  |
| DIAGNOSIS |  |  |  |  |  |  |
| **Statement #5: EoE is defined clinically by symptoms of esophageal dysfunction together with esophageal biopsies showing ≥15 eosinophils/hpf.** |  |  |  |  |  |  |
| **Statement #6: Common EoE symptoms and signs in younger children include feeding difficulties, abdominal pain, failure to thrive, and vomiting.** |  |  |  |  |  |  |
| **Statement #7: Common EoE symptoms in older children, adolescents and adults include dysphagia, excessive fluid intake with meals, avoidance of certain food textures, and food impaction.** | 8 | 7-9(2) | 9 | 3-9 | 28 (90.3%) | 0.537 |
| **Statement #8: Severe symptoms including transient food bolus impaction or dysphagia require immediate referral to gastroenterology (GI). Given frequent delays in accessing GI assessment in Canada, in cases with non-severe symptoms, it is reasonable to trial patients with possible EoE on high-dose PPI for symptomatic relief while waiting for consultation and endoscopy.** | 8 | 6.5-8(1.5) | 8 | 3-9 | 23 (74.2%) | 0.376 |
| **Statement #9: In situations where access to GI is prolonged or unavailable, for example in rural and remote regions, general surgeons may provide access to endoscopy and biopsy for EoE diagnosis and management. This endoscopy should follow the same standards outlined in this statement. Post-endoscopy, surgeons may collaborate with the allergist and gastroenterologist to coordinate management.** | 8 | 7.5-9(1.5) | 9 | 5-9 | 29 (93.5%) | 0.637 |
| **Statement #10: A trial of proton pump inhibitors is not required for diagnosis of EoE.** |  |  |  |  |  |  |
| **Statement #11: Biopsies from upper GI endoscopy are mandatory for diagnosis and, when possible, should be performed by an endoscopist. There should be ≥2 biopsies per level from at least two levels (distal, mid, and proximal esophagus), for a total of ≥4-6 biopsies. Biopsies of the stomach and duodenum should be considered [at the time of initial endoscopic exam] to rule out eosinophilic gastritis and duodenitis; especially in those with other foregut symptoms. Follow-up should be arranged with the Endoscopist to review the pathology results.** | 9 | 8-9(1) | 9 | 3-9 | 26 (83.9%) | 0.538 |
| **Statement #12: Endoscopic findings for EoE can include edema, rings, exudates, linear furrows, and stricture. They should be reported as EREFS including subscores. One can have increased eosinophils without macroscopic features.** | 9 | 8-9(1) | 9 | 5-9 | 29 (93.5%) | 0.672 |
| **Statement #13: In the event of a food impaction, if endoscopy is performed, taking esophageal biopsies at the same time are strongly encouraged so management can follow. High-dose PPI or topical corticosteroids should be trialled at this time.** | 8 | 7-9(2) | 9 | 2-9 | 25 (80.6%) | 0.379 |
| **Statement #14: Histology should be reported as eosinophil counts per 0.3mm2 (=per hpf), with EoE diagnosis requiring ≥15 eosinophils/hpf.** |  |  |  |  |  |  |
| **Statement #15: Classic features beyond esophageal eosinophilia can include dilated intercellular spaces, basal zone hyperplasia, eosinophilic abscesses as well as lamina propria fibrosis, and can strengthen a diagnosis.** |  |  |  |  |  |  |
| **Statement #16: The differential diagnosis of EoE includes gastroesophageal reflux disease (GERD), eosinophilic gastrointestinal disease (EGID), Crohn's disease, achalasia, hypermobility syndromes, drug hypersensitivity disorder, and others.** |  |  |  |  |  |  |
| **New Statements** |  |  |  |  |  |  |
| MANAGEMENT |  |  |  |  |  |  |
| **Statement #17: EoE provider care should be a collaborative effort between multiple providers (i.e. gastroenterologists, allergists, surgeons, pathologists, dietitians, and practitioners ) and includes shared decision-making with the patient.** | 9 | 8-9(1) | 9 | 6-9 | 30 (96.8%) | 0.700 |
| **Statement #18: It is important to transition care from paediatric to adult services to support them through this high risk period.** |  |  |  |  |  |  |
| **Statement #19: Initial management options for controlling symptoms, reducing inflammation, and preventing complications include PPI, empiric dietary elimination, and swallowed topical corticosteroids. If dilation is required, it must be used as complementary to other treatments as it will not control underlying inflammation.** | 9 | 8-9(1) | 9 | 7-9 | 31 (100%) | 0.804 |
| **Statement #20: After initiating treatment, repeat endoscopy and biopsy should occur anytime between 6 to 12 weeks after a change in management.** | 8 | 7.5-9(1.5) | 8 | 1-9 | 28 (90.3%) | 0.517 |
| **Statement #21: Mode of medication delivery (pill, dissolving medication, slurry or liquid, etc.) is a shared decision-making process between patient and provider to ensure adherence.** |  |  |  |  |  |  |
| **Statement #22: Empiric dietary elimination is best started with one (cow's milk) or two (cow's milk and wheat) food elimination, balancing efficacy, convenience, and adherence. Starting with a six-food elimination may result in higher rates of remission but is not recommended as first-line treatment due to limited convenience, poor adherence, impaired quality of life and other adverse outcomes.** | 9 | 8-9(1) | 9 | 6-9 | 29 (93.5%) | 0.724 |
| **Statement #23: Consultation with a dietitian is essential for patients on dietary elimination.** |  |  |  |  |  |  |
| **Statement #24: An elemental diet is rarely recommended for management of EoE due to poor taste, frequent need for enteral tube, high cost and significant impact on quality of life.** | 9 | 8-9(1) | 9 | 6-9 | 30 (96.8%) | 0.792 |
| **Statement #25: Patients with allergic conditions who have undergone prolonged and extensive dietary elimination for the treatment of EoE are at a heightened risk of developing IgE-mediated food allergies to the specific foods they have avoided.** | 8 | 8-9(1) | 9 | 5-9 | 28 (90.3%) | 0.630 |
| **Statement #26: Budesonide orodispersible tablet is approved by Health Canada for the treatment of EoE in adults. Off-label swallowed topical corticosteroid options include viscous budesonide and fluticasone MDI.** | 9 | 8-9(1) | 9 | 5-9 | 30 (96.8%) | 0.749 |
| **Statement #27: Systemic or long-term (e.g. oral prednisone) corticosteroids are not recommended for routine use in treatment of EoE.** | 9 | 8-9(1) | 9 | 4-9 | 28 (90.3%) | 0.652 |
| **Statement #28: Stricture development can be a complication of EoE. Some strictures may respond to medical treatment. But others may required dilation. While endoscopic dilation may alleviate symptoms, it is important to address disease control and stricture recurrence through aggressive treatment with antiinflammatory treatments.** | 8 | 8-9(1) | 9 | 4-9 | 28 (90.3%) | 0.561 |
| **Statement #29: Dupilumab is a Health Canada approved biologic for EoE and can be considered for severe disease and/or patients with multiple allergic conditions. Other biologics that are not approved by Health Canada have shown histologic improvement but persistence of symptoms in clinical trials.** | 8 | 7-9(2) | 9 | 3-9 | 25 (80.6%) | 0.437 |
| **Statement #30: The cost of Health Canada-licensed EoE medications can be high. Due to variable coverage by 3rd party prescription programs and lack of current coverage by provincial prescription drug plans, a coordinated effort is needed to ensure patients with EoE receive appropriate treatments regardless of location.** |  |  |  |  |  |  |
| **Statement #31: Allergy testing (skin prick tests, sIgE blood tests, or patch tests) to uncover food triggers of EoE is not recommended. Rather, if chosen, dietary elimination should be done empirically. The purpose of food allergy testing is to rule out potentially anaphylactic IgE-mediated food allergy when the history is suggestive of it.** |  |  |  |  |  |  |
| **Statement #32: The roles of an allergist in the management of EoE include selection and interpretation of skin prick or sIgE tests prior to re-introduction of previously eliminated foods, oral food challenges to rule out conversion to IgE-mediated food allergy, managing associated type-2 inflammatory conditions, and counselling and conducting allergen immunotherapy.** | 8 | 7-9(2) | 9 | 4-9 | 27 (87.1%) | 0.525 |
| **Statement #33: A subset of patients sensitized to pollen may experience seasonal intensification of EoE due to pollen allergy. Allergists can help patients distinguish between EoE and Pollen Food Allergy Syndrome to better manage symptoms.** | 9 | 8-9(1) | 9 | 5-9 | 29 (93.5%) | 0.620 |
| **Statement #34: It is unclear whether sublingual or oral immunotherapy causes or unmasks EoE, or whether the disease is simply associated with the therapy. Immunotherapy should be based on weighing benefits/risks and shared decision-making.** | 8 | 8-9(1) | 9 | 5-9 | 28 (90.3%) | 0.615 |
| **Statement #35: Recommended follow-up for uncontrolled EoE is every 3-4 months. In severe cases follow-up interval can be as short as 2-4 weeks. For well-controlled EoE recommended follow-up is 12-24 months.** | 8 | 8-9(1) | 9 | 3-9 | 27 (87.1%) | 0.554 |
| **Statement #36: Disease activity indices for symptomatic, endoscopic, histologic, and quality-of-life measures are available and can be used, although may not always be feasible to adopt in routine clinical settings.** | 8 | 8-9(1) | 8 | 5-9 | 28 (90.3%) | 0.656 |
| **Statement #37: Following disease state in EoE patients requires monitoring of symptoms and treatment adherence with endoscopic and histologic surveillance. Balancing these aspects requires shared decision making.** | 9 | 8-9(1) | 9 | 5-9 | 28 (90.3%) | 0.647 |
| **Statement #38: EoE has a negative impact on psychosocial status. Monitoring the overall well-being of EoE patients is an important part of follow-up.** |  |  |  |  |  |  |
| **Statement #39: The ultimate duration of therapy for patients who achieve control of their EoE is unclear in the literature. Given this is a long-term condition, the decision to continue treatment and in what form is dependent on severity of symptoms and disease, as well as shared decision-making with the patients and family; balancing risk and benefits of the treatment with the risks of complications (e.g. Stricturing disease).** | 8 | 8-9(1) | 9 | 6-9 | 29 (93.5% ) | 0.679 |
| **NEW: Statement #40: It is recommended to choose single interventions (e.g. diet or TCS, not both) when starting management. Multiple treatment modalities may require consultation with an EOE expert centre.** | 7 | 6-8(2) | 8 | 2-9 | 22 (71.0%) | 0.231 |
| **New statements** |  |  |  |  |  |  |

**Supplementary Table 4: Aggregate results of survey round 3**

| Recommendation | Median ranking | IQR | Mode | Absolute Range | n>=7 | Kappa |
| --- | --- | --- | --- | --- | --- | --- |
| DEFINITION |  |  |  |  |  |  |
| **Statement #1: EoE is a chronic inflammatory condition of the esophagus, characterized by eosinophilic inflammation. Pathophysiologic mechanisms include barrier dysfunction, delayed (non-IgE mediated) allergic responses to food (and or environmental allergens), type 2 inflammation and tissue remodelling.** |  |  |  |  |  |  |
| **Statement #2: EoE is increasing in prevalence, which can be partially attributed to increased awareness. The true prevalence is not well defined in Canada.** |  |  |  |  |  |  |
| **Statement #3: EoE occurs in all genders and ethnic backgrounds but is more commonly diagnosed in white males. It is often associated with allergic conditions such as IgE-mediated food allergy, asthma, atopic dermatitis, chronic rhinosinusitis with nasal polyps and allergic rhinitis. EoE can be associated with family history of EoE in first-degree relatives.** | 8 | 8-9 | 9 | 1-9 | 93.6% | 0.527 |
| **Statement #4: Long term complications of EoE include fibrosis and stricture development. These risks are increased with diagnostic delay and with untreated EoE.** |  |  |  |  |  |  |
| **New statements suggested** |  |  |  |  |  |  |
| DIAGNOSIS |  |  |  |  |  |  |
| **Statement #5: EoE is defined clinically by symptoms of esophageal dysfunction together with esophageal biopsies showing ≥15 eosinophils/hpf.** |  |  |  |  |  |  |
| **Statement #6: Common EoE symptoms in adolescents and adults include dysphagia and food impaction. Adaptive behaviours are frequently seen including drinking fluids to help swallow foods, cutting food into small pieces or pureeing, excessive chewing, prolonged mealtimes, avoiding hard textures and turning away tablets/pills.** | 9 | 8-9 | 9 | 7-9 | 100% | 0.752 |
| **Statement #7:Common EoE symptoms and signs in younger children additionally include feeding difficulties, abdominal pain, failure to thrive, and vomiting.** |  |  |  |  |  |  |
| **Statement #8: Severe symptoms such as food impaction with obstruction (inability to swallow secretions) need urgent care for endoscopic removal of food bolus. Taking esophageal biopsies at the same time as endoscopy is strongly encouraged. If food bolus impaction passes without need for emergency gastroscope, patients should be referred to GI for either new consultation or a follow-up appointment for endoscopic evaluation.** | 8 | 8-9 | 9 | 7-9 | 100% | 0.731 |
| **Statement #9: In situations where access to GI is delayed or unavailable, for example in rural and remote regions of Canada, general surgeons may provide access to endoscopy and biopsy for EoE diagnosis. This endoscopy should follow the same standards outlined in this statement. Post-endoscopy, surgeons may collaborate with the allergist and GI to coordinate management.** |  |  |  |  |  |  |
| **Statement #10: A trial of proton pump inhibitors is not required for diagnosis of EoE.** |  |  |  |  |  |  |
| **Statement #11: Endoscopic biopsies are required for the diagnosis of EoE, regardless of endoscopic appearance. There should be ≥2 biopsies per level from at least two segments (distal, mid, and proximal esophagus), for a total of ≥4-6 biopsies. Biopsies of the stomach and duodenum should be considered at initial endoscopic exam.** | 9 | 8-9 | 9 | 6-9 | 93.5% | 0.704 |
| **Statement #12:Endoscopic findings for EoE can include edema, rings, exudates, linear furrows, and stricture. Findings should be reported as EREFS including subscores. Macroscopic features are absent in some patients with increased eosinophils and are not required to make a diagnosis of EoE.** |  |  |  |  |  |  |
| **Statement #13: Histology should be reported as eosinophil counts per 0.3mm2 (=per hpf), with EoE diagnosis requiring ≥15 eosinophils/hpf.** |  |  |  |  |  |  |
| **Statement #14: Classic histologic features beyond esophageal eosinophilia can include dilated intercellular spaces, basal zone hyperplasia, eosinophilic abscesses as well as lamina propria fibrosis, and can strengthen a diagnosis.** |  |  |  |  |  |  |
| **Statement #15:The differential diagnosis of EoE includes gastroesophageal reflux disease (GERD), eosinophilic gastrointestinal disease (EGID), Crohn's disease, achalasia, hypermobility syndromes, drug hypersensitivity disorder, and others.** |  |  |  |  |  |  |
| **New Statements** |  |  |  |  |  |  |
| MANAGEMENT |  |  |  |  |  |  |
| **Statement #16: EoE care should be a collaborative effort between multiple providers (i.e. gastroenterologists, allergists, surgeons, pathologists, dietitians, and practitioners ) and includes shared decision-making with the patient.** |  |  |  |  |  |  |
| **Statement #17: It is important to transition care from paediatric to adult services to support patients through this high risk period.** |  |  |  |  |  |  |
| **Statement #18: Initial management options for controlling symptoms, reducing inflammation, and preventing complications include PPI, empiric dietary elimination, and swallowed topical corticosteroids. If dilation is required, it must be used as complementary to other treatments as it will not control underlying inflammation.** |  |  |  |  |  |  |
| **Statement #19: Empiric dietary elimination is best started with one (cow's milk) or two (cow's milk and wheat) food elimination, balancing efficacy, convenience, and adherence. Starting with a six-food elimination may result in higher rates of remission but is not recommended as first-line treatment due to limited convenience, poor adherence, impaired quality of life and other adverse outcomes.** |  |  |  |  |  |  |
| **Statement #20: Consultation with a dietitian is essential for patients on dietary elimination.** |  |  |  |  |  |  |
| **Statement #21: An elemental diet is rarely recommended for management of EoE due to poor taste, frequent need for enteral tube, high cost and significant impact on quality of life.** |  |  |  |  |  |  |
| **Statement #22: Patients with allergic conditions who have undergone prolonged and extensive dietary elimination for the treatment of EoE are at a heightened risk of developing IgE-mediated food allergies to the specific foods they have avoided.** |  |  |  |  |  |  |
| **Statement #23: Mode of medication delivery is a shared decision-making process between patient and provider to ensure adherence but prescribers should recognize that patients with EoE frequently have challenges with pills such as tablets and capsules.** |  |  |  |  |  |  |
| **Statement #24: Systemic or long-term corticosteroids (e.g. oral prednisone) are not recommended for routine use in treatment of EoE.** |  |  |  |  |  |  |
| **Statement #25: Budesonide orodispersible tablet is approved by Health Canada for the treatment of EoE in adults. Off-label swallowed topical corticosteroid options include viscous budesonide and fluticasone MDI.** |  |  |  |  |  |  |
| **Statement #26: Dupilumab (anti IL-4/13) is a Health Canada approved biologic for EoE and can be considered for disease (1) refractory to conventional treatments; or (2) those experiencing side effects with conventional therapy; (3) and/or patients with currently approved concurrent severe allergic conditions.** | 8 | 8-9 | 9 | 3-9 | 90.3 | 0.602 |
| **Statement #27: The cost of Health Canada-licensed EoE medications can be high. Due to variable coverage by 3rd party prescription programs and lack of current coverage by provincial prescription drug plans, a coordinated effort is needed to ensure patients with EoE receive appropriate treatments regardless of location.** |  |  |  |  |  |  |
| **Statement #28: Development of luminal narrowing and/or stricture can be a complication of EoE. Some narrowing may respond to medical treatment while others may require dilation. While endoscopic dilation may alleviate symptoms, it is important to address mucosal inflammation and prevent recurrence of narrowing through treatment with anti-inflammatory therapies.** | 9 | 8-9 | 9 | 6-9 | 96.8 | 0.763 |
| **Statement #29: Allergy testing (skin prick tests, sIgE blood tests, or patch tests) to uncover food triggers of EoE is not recommended. Rather, if chosen, dietary elimination should be done empirically. The purpose of food allergy testing is to rule out potentially anaphylactic IgE-mediated food allergy when the history is suggestive of it.** |  |  |  |  |  |  |
| **Statement #30: In addition to shared understanding and experience in the pathophysiology and management of EoE with gastroenterologists, allergists have unique experience in their ability to manage concurrent and complicating allergic conditions, such as IgE-mediated food allergy and determination of relevant aeroallergen sensitization.** | 8 | 7.5-9 | 9 | 5-9 | 90.3 | 0.595 |
| **Statement #31: A subset of patients sensitized to pollen may experience seasonal intensification of EoE due to pollen allergy. Allergists can help patients distinguish between EoE and Pollen Food Allergy Syndrome to better manage symptoms.** |  |  |  |  |  |  |
| **Statement #32: It is unclear whether sublingual or oral immunotherapy causes or unmasks EoE, or whether the disease is simply associated with the therapy. Immunotherapy should be based on weighing benefits/risks and shared decision-making.** |  |  |  |  |  |  |
| **Statement #33: When feasible, single medical interventions or monotherapy (Diet/STC) should be evaluated in isolation to know their effectiveness. Combination therapies may be considered for more severe diseases.** | 8 | 7.5-9 | 9 | 6-9 | 90.3 | 0.614 |
| **Statement #34: Clinical evaluation alone is not sufficient to assess treatment efficacy. Repeat endoscopy and biopsy to assess treatment efficacy after a change in management ideally should occur at 6-12 weeks.** | 9 | 8-9 | 9 | 5-9 | 87.1 | 0.598 |
| **Statement #35: It is important to ensure follow-up of EoE. The interval for follow up including endoscopy may vary depending on symptoms (frequency and severity), phenotype including history of strictures, and amount of inflammation, with uncontrolled EoE requiring more frequent follow-up.** | 8 | 8-9 | 9 | 6-9 | 90.3 | 0.649 |
| **Statement #36: Disease activity indices for symptomatic, endoscopic, histologic, and quality-of-life measures are available and can be used, although may not always be feasible to adopt in routine clinical settings.** |  |  |  |  |  |  |
| **Statement #37: EoE has a negative impact on psychosocial status. Monitoring the overall well-being of EoE patients is an important part of follow-up.** |  |  |  |  |  |  |
| **Statement #38: The ultimate duration of therapy for patients who achieve control of their EoE is unclear in the literature. Given this is a long-term condition, the decision to continue treatment and in what form is dependent on severity of symptoms and disease, as well as shared decision-making with the patients and family; balancing risk and benefits of the treatment with the risks of complications (e.g. Stricturing disease).** |  |  |  |  |  |  |
| **New statements** |  |  |  |  |  |  |
